# Pancreatic endocrine and exocrine signaling and crosstalk in physiological and pathological status

**DOI:** 10.1038/s41392-024-02098-3

**Published:** 2025-02-14

**Authors:** Chenglin Hu, Yuan Chen, Xinpeng Yin, Ruiyuan Xu, Chenxue Yin, Chengcheng Wang, Yupei Zhao

**Affiliations:** 1 Department of General Surgery, Peking Union Medical College Hospital, Peking Union Medical College, Chinese Academy of Medical Sciences, Beijing, PR China; 2 Key Laboratory of Research in Pancreatic Tumor, Chinese Academy of Medical Sciences, Beijing, PR China; 3 State Key Laboratory of Complex, Severe, and Rare Diseases, Peking Union Medical College Hospital, Chinese Academy of Medical Science and Peking Union Medical College, Beijing, PR China; 4 National Infrastructures for Translational Medicine, Peking Union Medical College Hospital, Beijing, PR China; 5 Institute of Clinical Medicine, Peking Union Medical College Hospital, Beijing, PR China

**Keywords:** Gastrointestinal cancer, Metabolic disorders

## Abstract

The pancreas, an organ with dual functions, regulates blood glucose levels through the endocrine system by secreting hormones such as insulin and glucagon. It also aids digestion through the exocrine system by secreting digestive enzymes. Complex interactions and signaling mechanisms between the endocrine and exocrine functions of the pancreas play a crucial role in maintaining metabolic homeostasis and overall health. Compelling evidence indicates direct and indirect crosstalk between the endocrine and exocrine parts, influencing the development of diseases affecting both. From a developmental perspective, the exocrine and endocrine parts share the same origin—the “tip-trunk” domain. In certain circumstances, pancreatic exocrine cells may transdifferentiate into endocrine-like cells, such as insulin-secreting cells. Additionally, several pancreatic diseases, including pancreatic cancer, pancreatitis, and diabetes, exhibit potential relevance to both endocrine and exocrine functions. Endocrine cells may communicate with exocrine cells directly through cytokines or indirectly by regulating the immune microenvironment. This crosstalk affects the onset and progression of these diseases. This review summarizes the history and milestones of findings related to the exocrine and endocrine pancreas, their embryonic development, phenotypic transformations, signaling roles in health and disease, the endocrine-exocrine crosstalk from the perspective of diseases, and potential therapeutic targets. Elucidating the regulatory mechanisms of pancreatic endocrine and exocrine signaling and provide novel insights for the understanding and treatment of diseases.

## Introduction

The pancreas is a unique organ with dual functions, both endocrine and exocrine. The endocrine portion of the pancreas consists of islets, which help control blood glucose levels by releasing hormones like insulin, glucagon, and somatostatin. Each hormone functions through a specific signaling pathway, not only regulates endocrine and exocrine functions but also plays a key role in the homeostasis of cells. At the same time, the exocrine part assists in the breakdown of carbohydrates, proteins, and fats by secreting a variety of enzymes.^1^ Historically, these two functions have often been studied independently. However, emerging evidence indicates that intricate crosstalk between the endocrine and exocrine components plays a crucial role in maintaining pancreatic function and has significant implications for various diseases.

Recent research highlights the importance of endocrine-exocrine crosstalk in the pathogenesis of serious conditions like pancreatic cancer and diabetes. Pancreatic ductal adenocarcinoma (PDAC), a highly aggressive cancer, is often associated with endocrine dysfunctions such as diabetes.^2^ In the case of PDAC, this crosstalk becomes particularly evident. Notably, many PDAC patients develop new-onset diabetes as an early symptom, likely due to the disruption of insulin secretion caused by tumor growth. In turn, diabetes, particularly long-standing type 2 diabetes (T2D), is recognized as a risk factor for PDAC development.^3^ Similarly, chronic pancreatitis, another exocrine-related disorder, can lead to both exocrine and endocrine insufficiencies, further demonstrating the bidirectional influence between these systems.^4^ Understanding these interactions is crucial, as both PDAC and diabetes are not only common but also deadly, underscoring the need for comprehensive research that explores how dysfunction in one system can impact the other. The crosstalk between endocrine and exocrine functions involves complex signaling pathways, such as the insulin-glucagon feedback loop, which plays a crucial role in regulating both glucose metabolism and exocrine enzyme secretion. Disruption in these pathways, as seen in PDAC and chronic pancreatitis, can exacerbate disease progression by further impairing both metabolic and digestive functions. However, despite its significance, this potential crosstalk remains underappreciated in many studies, with the majority of research focusing on either the endocrine or exocrine aspects in isolation. This oversight may lead to an incomplete understanding of pancreatic function and its involvement in disease progression.

This review aims to bridge this gap by systematically examining the cross-functional interactions between the endocrine and exocrine components of the pancreas. By highlighting key studies that have explored the signaling of pancreatic endocrine and exocrine components and crosstalk, we seek to provide a comprehensive overview of how this signaling pathway as well as interactions contribute to the development of both physiological and pathological conditions such as diabetes, pancreatitis, and PDAC. Furthermore, this review also highlights the need for an integrative approach to research. Future studies should focus on exploring both endocrine and exocrine dynamics simultaneously to better understand the complex mechanisms underlying diseases.

## Historical discoveries and milestone events in pancreatic research

### Islets and cells in islets

In 1869, Paul Langerhans, a German pathologist, first identified clusters of cells within the pancreas while studying the histology of the organ. These clusters, later known as the islets of Langerhans, were described as distinct from the surrounding acinar cells responsible for exocrine functions.^5^ Although Langerhans did not hypothesize their specific function, his discovery laid the groundwork for future investigations into their role in endocrine regulation.

In 1893, Gustave-Édouard Laguesse, building on Langerhans’ work, suggested that these islets might serve an endocrine function.^6^ This hypothesis marked the beginning of focused research into the hormonal roles of the pancreas, particularly in the regulation of blood glucose levels. In the following decades, researchers connected the function of the islets of Langerhans with a substance crucial for glucose metabolism.

In 1921, Banting and Best’s isolation of insulin allowed for its purification and subsequent clinical testing.^7^ Before the discovery of insulin, diabetes was often fatal, but the ability to administer insulin transformed the disease into a manageable chronic condition, significantly improving the quality of life and life expectancy for millions of patients.

The diversity of pancreatic islet cells was first described in 1907 by Lane, who categorized them into A cells (α cells) and B cells (β cells).^8^ In 1923, Kim and Murlin discovered glucagon, a substance that induces hyperglycemia.^9^ The source of glucagon remained unknown until 1962 when α cells were identified as its origin. Further studies by Bellman et al. refined this understanding by distinguishing A1 cells and A2 cells, with A2 cells being the glucagon-producing α cells.^10–12^ D cells (δ cells), which were different from the previously described A and B cells, were first identified by Bloom in 1931.^13^ Somatostatin, the hormone produced by D cells, was later discovered in 1975.^13–15^ Pancreatic polypeptide (PP) was initially found during the isolation of insulin in chickens in 1968,^16^ and its localization in a new islet cell type, PP cells, was confirmed in chickens in 1974 and later in human islet.^17,18^

### Enzymes and acinar cells

The study of the exocrine part of the pancreas can be traced back to 1856. Claude Bernard, a famous French physiologist, played a pivotal role in advancing knowledge about the pancreas’ function in digestion. In 1856, Bernard discovered that the pancreas secreted a fluid capable of emulsifying fats. This fluid, now known as pancreatic juice, contains enzymes critical for the digestion of fats, proteins, and carbohydrates.^19^ His work laid the groundwork for the study of the pancreas's exocrine function, highlighting its crucial role in the digestive system. His discovery marked the first step in understanding how the pancreas contributes to digestion beyond its previously known endocrine functions. Trypsin was first isolated in 1876 by the German scientist Willy Kuhne, who was the first to observe alterations in the secretion of pancreatic acinar cells under a microscope.^20^ Ivan Pavlov, a Russian physiologist, conducted extensive studies on the digestive system, focusing on the regulatory mechanisms of pancreatic enzyme secretion. Through his classical conditioning experiments in the 1890s, Pavlov demonstrated the neural regulation of pancreatic secretion, showing how stimuli such as the sight and smell of food could trigger enzyme release. Pavlov’s research found the regulation of the pancreatic exocrine function of the nervous system, making an important contribution to understanding the pancreatic enzyme secretion mechanisms.^21,22^ He was awarded the Nobel Prize in Physiology or Medicine for his significant work in understanding the mechanisms of digestion.

Acinar cells are the main executors of pancreatic exocrine function. In the mid-20th century, George E. Palade used electron microscopy to study the ultrastructure of pancreatic acinar cells responsible for producing and secreting digestive enzymes. In 1974, he was honored with the Nobel Prize in Physiology or Medicine for his groundbreaking discoveries related to the cell’s structural and functional organization.

**Fig. 1:**
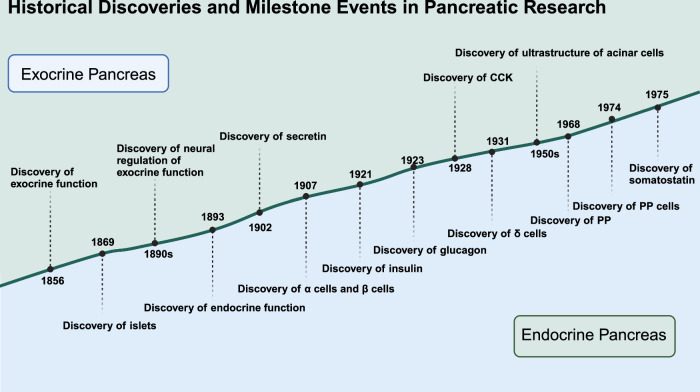
Historical discoveries and milestone events in pancreatic research. Significant historical milestones in the understanding of pancreatic structure and function highlight the discovery timeline of various components and functions of the pancreas. Created in BioRender.com

We can appreciate how early discoveries laid a robust foundation for the in-depth study of the pancreas’ exocrine functions and their critical role in digestion (Fig. 1). This understanding not only enriched basic biological knowledge but also drove significant clinical innovations that continue to benefit patients with pancreatic and digestive disorders in the following decades. Future studies could further explore this relationship, potentially leading to more comprehensive treatment strategies that address both digestive and metabolic disorders simultaneously. By continuing to build on these foundational discoveries, we can develop more targeted therapies that not only treat the symptoms of pancreatic diseases but also address their root causes.

### Endocrine and exocrine signaling molecules

Except for the discovery of insulin, the discovery of insulin receptors made great contributions to understanding the underlying signaling mechanisms of how insulin works in glucose metabolism. In 1971, two research teams identified the insulin receptor by observing the binding of ^125^I-labeled insulin to the membranes of adipose tissue and liver cells.^23,24^ And in the following few years, the interactions between insulin and insulin receptors in various healthy or pathological states, like obesity and diabetes, had been elucidated.^25–30^

Glucagon produced by the α cells in the pancreas increases blood glucose levels by promoting glycogen breakdown and gluconeogenesis in the liver. Early in 1966, its function had been regarded as a counter-regulatory hormone that complements insulin’s actions, maintaining glucose homeostasis.^31^

Somatostatin was identified in 1975, produced by δ cells in the pancreas, and demonstrated its inhibitory effects on the secretion of both insulin and glucagon.^13–15^ Within the islets of Langerhans, this hormone functions as a paracrine regulator, fine-tuning the balance of glucose-regulating hormones.^32,33^

Pancreatic polypeptide, produced by PP cells, was found to influence both endocrine and exocrine pancreatic functions. It modulates gastrointestinal motility and the release of digestive enzymes, linking the endocrine and exocrine functions of the pancreas.^34^

In 1902, Bayliss and Starling discovered secretin, the first hormone identified, which stimulates the pancreas to secrete bicarbonate-rich fluid.^35,36^ This neutralizes stomach acid, providing an optimal pH for digestive enzyme activity in the intestine.^37^ As to the digestive enzymes regulator, cholecystokinin was discovered by Ivy and Oldberg in 1928, stimulating the release of pancreatic digestive enzymes and bile from the gallbladder, aiding in the digestion of fats and proteins.^38^ These hormones exemplify the intricate regulatory mechanisms governing exocrine pancreatic secretion.^39,40^

The pancreas’ ability to regulate both endocrine and exocrine functions through these signaling molecules demonstrates its integrated role in maintaining overall metabolic homeostasis. This dual functionality is crucial for efficient nutrient digestion and glucose metabolism. These discoveries underscore the importance of crosstalk and feedback mechanisms in the pancreas.

## The close connection between endocrine and exocrine parts of the pancreas

### Pancreas development

In the process of embryonic development, pancreatic cells of all types derive from the foregut endoderm. In mice, pancreatic development initiates at E8.5. The dorsal endoderm thickens to form a bud with a stalk, known as the dorsal pancreatic bud.^41^ Concurrently, the ventral pancreatic bud emerges as the endodermal epithelium proliferates outward from the ventral end of the foregut. The notochord inhibits the sonic hedgehog, allowing the dorsal pancreatic bud to develop.^42^ These structures are surrounded by mesenchyme. The transcription factor Pdx1 (pancreatic and duodenal homeobox 1) is considered the earliest (E8.5) and most specific gene expressed in embryonic pancreatic development.^43^ Pdx1 plays a crucial role not only in the early expression of embryonic pancreatic development but also in the differentiation of endocrine precursor cells into insulin-secreting cells. In mice, Pdx1 inactivation in the mature pancreas leads to diabetes, underscoring its role in maintaining normal pancreatic function.^44^

At E9.5, Pdx1 is highly expressed in the cells of both dorsal and ventral pancreatic bud.^45^ The pancreatic bud grows outward to form a curled structure with a hollow center. Simultaneously, the dorsal pancreatic epithelium separates from the notochord in the region of high Pdx1 expression. Endothelial cells from the dorsal aorta come into contact with the dorsal pancreatic endoderm, providing metabolic support and a range of inductive signals for pancreatic development.^46^ Subsequently, the dorsal aorta separates from the dorsal pancreatic epithelium, which becomes enveloped by mesenchyme. Mesenchyme is crucial for pancreatic development and can secrete fibroblast growth factor 7 (FGF7) and FGF10 to promote embryonic epithelial proliferation.^47^ Mice with Pdx1 deficiency exhibit pancreatic epithelial cells that fail to respond to growth signals from mesenchyme.^48^ At E9.5, in addition to Pdx1, the pancreatic epithelial precursor cells also express pancreas-specific transcription factors, including Ptf1a, SOX9, Nkx2.2, Nkx6.1, and Nkx6.2. These transcription factors regulate pancreatic development and fate determination, and their co-expression characterizes pancreatic epithelial progenitor cells.^45^

After E10.5, pancreatic epithelial progenitor cells undergo extensive proliferation and expansion. Simultaneously, the intestinal tube rotates, and the ventral and dorsal pancreatic buds merge to form branched structures with distinct differentiation potentials in the tip and trunk domains. The formation of these branched structures may be facilitated with the assistance of the Rho-GTPase family member Cdc42.^49^ Cells in the tip domain are Ptf1a^+^ multipotent progenitor cells (MPCs) capable of generating pancreatic endocrine cells, ductal cells, and acinar cells. Stem cells in the trunk domain, identified by Nkx6 expression, can differentiate into ductal cells and endocrine cells. By E13, MPCs in the tip domain lose their multipotency and become acinar precursors, capable only of producing acinar cells. Cell fate specificity and pancreatic morphogenesis are closely tied to cell-cell interactions and the regulatory effects of surrounding tissues, including blood vessels and mesenchyme.^50^

The final event in islet morphogenesis is the differentiation of endocrine cells. studies have shown that Ngn3^+^ endocrine progenitor cells are unipotent precursors, distinct from ductal progenitors, and can differentiate into five endocrine cell types.^51,52^ Ngn3, which is uniquely expressed in endocrine cells is critical for the differentiation of endocrine cells.^53^ Various transcription factors play a role in the differentiation process, leading to the formation of distinct endocrine cell types.^54^ α cell transcription factors include Foxa2, Nkx2.2, Pax6, and Arx. In contrast, β cell differentiation requires the expression of Mafb, Pdx1, Hlxb9, Pax4, Pax6, Islet1, Nkx2.2, and Nkx6.1.

Endocrine cells detach from the ductal epithelium, and migrate toward the mesenchyme and blood vessels, ultimately forming islets. Although this process is completed after E13, single-cell RT-PCR can detect the coexistence of glucagon, insulin, somatostatin, and pancreatic polypeptide in the dorsal pancreatic epithelium as early as E10.5.^55^ Co-expression of glucagon and insulin can be detected at E9.5, suggesting that these co-expressing cells may be precursors of certain endocrine cells.^56^ However, the fate of double-hormone-positive cells is still unclear, as studies have shown that the development of α and β cells occurs independently of each other.^57–59^

SOX9 plays a crucial role in pancreatic duct development and is currently recognized as one of the markers for the ductal lineage.^60^ It is expressed in pancreatic progenitor cells and plays a role in maintaining the progenitor cell population. Simultaneously, SOX9 promotes the expression of Ngn3, thereby stimulating the generation of endocrine progenitor cells.^61^ Following the activation of Ngn3, SOX9 expression is subsequently downregulated to facilitate the differentiation of endocrine cells by modulating the Notch pathway’s activity gradient.^62^ Under high levels of Notch signaling, Hes1 (Ngn3 inhibitory factor) is induced, leading to the retention of SOX9 expression in progenitor cells, which then transition into ductal cells. Ductal cells possess the potential to transdifferentiate into endocrine cells.

Currently, research on pancreatic development primarily focuses on murine models. Due to the genetic similarity between mice and humans, as well as the ease of experimentation, mice are commonly used in studies related to pancreatic development. While the murine model provides critical insights into pancreatic development, it is important to recognize the similarities and differences in the human context. Limited by ethical requirements for embryological research in humans, our understanding of human pancreatic development is far less comprehensive than that of mice. However, we can still discern similarities and differences between human and mouse pancreatic development from existing studies.

In terms of commonalities, both human and mouse pancreatic development follow a similar developmental pattern, including the formation of dorsal and ventral pancreatic buds and the expression of certain genes that drive a series of differentiation processes. This has been thoroughly reviewed by Rachael et al.^63^ Important genes in mouse pancreatic development, such as SOX9, Ptf1a, and Ngn3, are also present in the differentiation of pancreatic cells in humans.^64–66^

However, there are significant differences in the timeline and gene expression patterns between human and mouse pancreatic development. First, the developmental timeline of the human pancreas is significantly longer than that of the mouse. Pancreatic development in mice begins around embryonic day 8.5 (E8.5) and most organ differentiation is completed by around E14.^45^ In contrast, while the initiation of pancreatic development in humans is similar, the process of maturation takes much longer, with full pancreatic maturation only achieved in the mid-to-late stages of pregnancy.^65^

Gene expression patterns also differ between species. For example, the Pdx1 gene is strongly expressed early in the pancreatic development of mice (E8.5) and remains at high levels throughout the differentiation process.^43^ In humans, while Pdx1 is also expressed early on, the timing and intensity of its expression may be influenced by more complex regulatory mechanisms, potentially leading to differences in the progression of pancreatic development between humans and mice. Mice with Pdx1 loss exhibit pancreatic hypoplasia, a phenomenon confirmed over 30 years ago.^67^ In 1997, a case was reported of a newborn with homozygous loss of Pdx1 who developed diabetes, presenting with exocrine insufficiency and hyperglycemia.^68^ In humans, mutations in the Pdx1 gene have been associated with neonatal diabetes, often accompanied by varying levels of exocrine dysfunction.^69^ Heterozygous mutations in Pdx1 seem to have a negative impact on insulin-positive cells,^70^ a phenomenon also observed in mice, where Pdx1 heterozygous mutations result in impaired β cell function and increased apoptosis.^71,72^

The expression of the Ngn3 gene also shows different timing and patterns in the two species. In mice, Ngn3 expression is concentrated between E13.5 and E15.5, while in humans, the peak expression occurs later, correlating with the later maturation of human islet cells.^73^ Mutations in the Ngn3 gene affecting pancreatic and intestinal endocrine cell differentiation have been reported,^74^ but the manifestations in humans are inconsistent. Some studies have found that individuals carrying completely inactivated Ngn3 mutations do not develop permanent neonatal diabetes.^75^ Early research suggested that Ngn3 insufficiency might be compensated by other mechanisms,^76^ but subsequent studies revealed that these mutations might be functionally hypomorphic rather than fully inactive.^77^ Furthermore, even with only 10% of Ngn3 functionality, pancreatic endocrine cell differentiation can still occur.^78^ The reasons behind the differing pancreatic functions following Ngn3 inactivation in humans and mice remain to be explored.

Overall, the differences between human and mouse pancreatic development may partially explain the specificities and disease susceptibilities in human pancreatic development, particularly when it comes to single nucleotide polymorphisms (SNPs) in key genes, which may lead to developmental failures or increased disease risk. A meta-analysis of SNPs in T2D identified overlaps with a range of pancreatic development genes.^79^ Therefore, understanding and comparing these interspecies differences is crucial for research on related diseases such as T2D, neonatal diabetes, and pancreatic hypoplasia.

The development of the pancreatic endocrine and exocrine parts is not mutually exclusive. All pancreatic cells originate from pancreatic progenitor cells, and this shared cellular origin may constitute a critical foundation for the crosstalk between the endocrine and exocrine parts. This common lineage suggests that disruptions or alterations in the developmental pathways could have widespread effects across both systems, potentially leading to a variety of pancreatic diseases. The crosstalk between these parts is not only structural but also functional, as signaling molecules and metabolic processes within the exocrine pancreas can influence endocrine function, and vice versa. Understanding this interplay opens up new avenues for research, particularly in exploring how early developmental signals might be manipulated to prevent or treat diseases like diabetes and pancreatitis.

### Transdifferentiation of pancreatic exocrine cells into endocrine cells

**Fig. 2:**
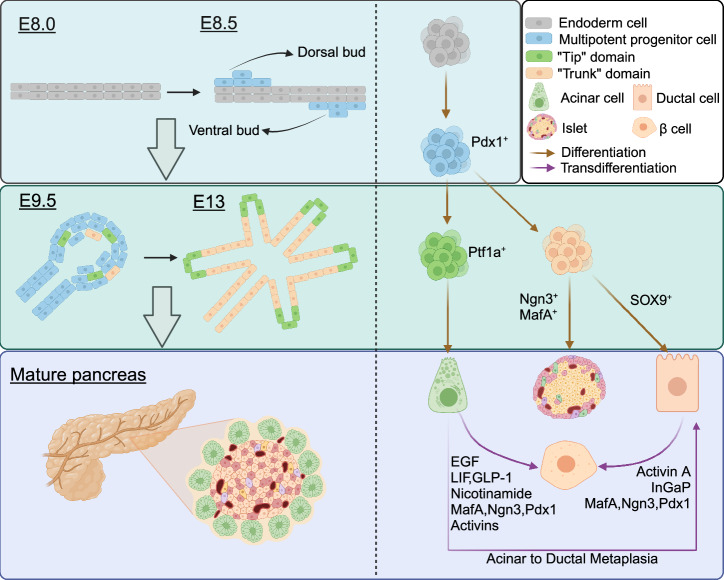
Pancreas development, cell differentiation, and transdifferentiation. At E8.5, the dorsal and ventral endoderm thickens to form two buds, called dorsal buds and ventral buds respectively. Cells in the two buds are Pdx1^+^ multipotent progenitor cells. At E9.5, the curled structure forms. Subsequently, these multipotent stem cells differentiate into two domains that together form a ramified structure. “Tip” domain cells differentiated into Ptf1a^+^ acinar precursor cells, while “Trunk” domain cells differentiated into SOX9^+^ duct precursor cells and Ngn3^+^ endocrine precursor cells. Transdifferentiation of acinar cells and ductal cells into islet endocrine cells and ADM can occur in mature pancreas under specific conditions. Created in BioRender.com

Transdifferentiation of pancreatic cells is the process by which a differentiated cell is converted into another cell, although it was once believed that the cellular phenotype of a mature somatic cell cannot be changed. However, an accumulating number of studies have proved that pancreatic cells are highly plastic. This process involves cellular reprogramming, such as β cell neogenesis, where new β cells regenerate from alternative pancreatic progenitors in the adult pancreas. A notable example of pancreatic transdifferentiation is acinar ductal metaplasia (ADM). Under certain conditions, pancreatic exocrine cells, including ductal and acinar cells, possess the ability to transdifferentiate into insulin-secreting cells (Fig. 2).

Acinar cells are highly differentiated cells while they are highly plastic. Recent studies have shown that acinar cells are able to regenerate β cells, although longer processes are required.^80^ However, in adult humans and mice, β cell regeneration assays showed that newly generated β cells were derived from the original β cells,^81,82^ and do not involve the corresponding progenitor cells.^83^ And the process of transdifferentiation can be completed only before birth.^84,85^ In vitro, rat acinar cells could be transdifferentiated into insulin-secreting cells when cultured with EGF and LIF,^86^ or with a suspension of EGF and nicotinamide. The lineage tracing system found that neonatal cells were derived from acinar cells expressing amylase and elastase. Enzymatic dissociation of pancreatic acini triggers EGF signaling, and blocking the EGF receptor kinase inhibits transdifferentiation. These newly formed cells can secrete insulin in response to glucose and other secretagogues, though their secretory capacity is lower than that of native β cells,^87^ likely due to the limited progenitor pool. Rat acinar cells AR42J were transformed into endocrine cells by treatment with GLP-1, exendin-4, or activins.^88–90^ In vivo, combinations of different transcripts can transdifferentiate acinar cells into three different endocrine cell subtypes (β cells, α cells, and δ cells),^91^ suggesting that transcription factors that promote endocrine cell fate predominate over the acinar cell program. Studies have shown that three transcription factors, Ngn3, Pdx1, and MafA can reprogram differentiated pancreatic exocrine cells in adult mice into cells that closely resemble β cells.^92^ The process depends on the magnitude of expression of the three transcription factors and the reprogramming-induced inflammatory response.^93^ Interestingly, Chen et al. found that intestinal crypt cells could even differentiate into β cells with the participation of these three transcription factors.^94^ This transdifferentiation ability is not entirely unexpected, as intestinal stem cells possess multipotency, meaning they can differentiate into various cell types, including endocrine cells. However, this study demonstrates that under appropriate conditions, intestinal cells can be directed to transform into β cells, providing new possibilities for cell replacement therapies in diabetes treatment. This also indicates that transcription factors not only play a role in the early stages of cell fate determination but can also induce mature cells to re-enter a differentiation state under certain conditions and redirect their differentiation pathway. This phenomenon indicates the potential of cellular plasticity and opens new avenues for regenerative medicine and disease treatment. The combination of the transcription factors Ngn3, Pdx1, MafA, and Pax4 can induce morphological changes and enhance insulin gene expression in AR42J cells.^95^ Insm1 further enhances endocrine transdifferentiation in the AR42J cell line by increasing the number and intensity of simultaneously activated ITFs and MafA in insulin-positive cells.^96^ The transcription factor-driven conversion of adult acinar cells is inhibited by Notch1 and Hedge signaling pathways.^97^ Activated Notch1 signaling prevents re-expression of Ngn3.^98^

Ductal cells that are more closely apposed along the path of embryonic development can also transform into islet cells under a variety of conditions. This outcome was experimentally accomplished through pancreatic duct ligation in mice and pancreatectomy in rats.^99,100^ The loss of acinar and islet cells indicates that ductal cells have the potential to differentiate into both acinar and endocrine cell types.^101^ Ductal cells in the adult pancreas exhibit a potential ability to generate β cells. Retrograde pancreatic ductal injection of an adenoviral vector facilitates gene transduction, resulting in the reprogramming of ductal cells into β cells and promoting the proliferation of existing β cells.^102^ Disruption and remodeling of cadherin-mediated cell-cell adhesion is crucial in the transdifferentiation of pancreatic ductal cells into insulin-secreting cells,^103^ in which PI3K has taken a vital part.^104^ When hESC-derived ductal cells were prompted to undergo partial epithelial-mesenchymal transition (EMT) by activin A treatment under hypoxia, the resulting cells showed significant expression of key endocrine markers, especially those associated with β cells.^105^ In the mouse model of acute pancreatitis, only a small number of Krt5+ positive cells had the ability to differentiate into beta cells. And this differentiation is dependent on the inhibition of Notch signaling.^106^ Notably, transcription factors Ngn3, MafA, and Pdx1 are similarly known to be important during endocrine transdifferentiation of ductal cells. In the adult pancreas, Ngn3 expression is typically low but increases during β cell neogenesis triggered by pancreatic duct ligation.^107^ The most notable outcome following prolonged misexpression of Ngn3 in adult duct cells is a substantial and continuous increase in the number of all islet cell types. Lineage tracing experiments indicate that newly generated β cells arise from the Ngn3 expressing ductal epithelium.^108^ Whether acinar or ductal cells, activation of Ngn3 has been implicated in the transformation into endocrine cells, which subsequently induce the expression of endocrine-related genes.^109^

Transdifferentiation of exocrine cells into exocrine cells, such as ADM, and mutual transdifferentiation of endocrine cells were reported in previous studies.^110,111^ In this review, we mainly focus on the interaction between the endocrine and exocrine parts of the pancreas. Transdifferentiation of pancreatic exocrine cells reveals that endocrine and exocrine cells of the pancreas are not mutually independent of one another in their cellular phenotype and fate. Although research in pancreatic cell transdifferentiation has made certain progress, there is sufficient evidence that pancreatic acinar and ductal cells have the potential ability to become cell replacement therapies for some diseases. Under specific conditions, these cells have the ability to differentiate into various functional pancreatic cell types, including islet and acinar cells. These stem-like pancreatic cells have potential in regenerative medicine because they may be used to repair or replace damaged pancreatic tissue, especially in diabetes or chronic pancreatitis. Scientists are working to reveal the population of stem cells or pluripotent progenitor cells within the pancreas, understand how they are activated in disease states, and how these cells can be harnessed for therapy. However, due to the differences between species existing in rodents and humans, it is difficult for animal experimental models to achieve clinical translation in humans, so more researches are needed to explore the regenerative mechanisms of transdifferentiation and more potential alternative sources of β cells.

## Pancreatic endocrine signaling

**Fig. 3:**
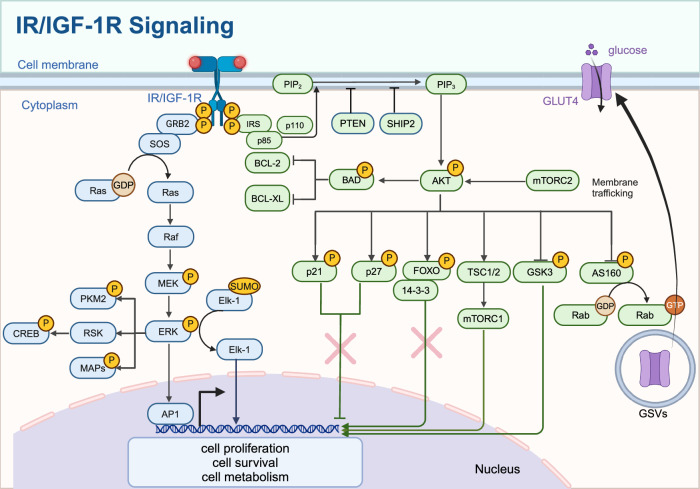
IR/IGF-1R signaling. The binding of insulin or IGF-1 to their receptors activates IRS proteins, leading to the activation of PI3K (p85/p110) and subsequent conversion of PIP2 to PIP3. Activation of AKT, which phosphorylates targets such as TSC1/2, mTORC1, AS160, and GSK3, regulating glucose uptake via GLUT4 translocation, protein synthesis, and cell survival. The Ras/MAPK pathway is activated through GRB2 and SOS, leading to the activation of RAF, MEK, and ERK, influencing cell proliferation and differentiation. The nuclear effects of these pathways include the regulation of transcription factors like FOXO, CREB, and Elk-1, affecting gene expression related to cell cycle, apoptosis, and metabolism. Created in BioRender.com

Endocrine signal refers to the hormones secreted by endocrine cells, through the circulation of the blood passed to the target cells or organs, regulating physiological function. Pancreatic endocrine signaling plays a key role not only in the regulation of blood glucose, metabolism, and homeostasis but also in various cell life activities^112–115^ (Fig. 3).

### Insulin and insulin receptor

The circulating form of insulin (activated form) is made of two chains, A chain and B chain, which consist of 21 amino acids and 30 amino acids, respectively, linked by two disulfide bonds.^116^ Insulin is converted from proinsulin in pancreatic β cells. In the Golgi apparatus, proinsulin is processed into insulin and C-peptide, which are stored in secretory granules and released when blood glucose levels rise.^117^

The insulin receptor (IR) belongs to the (αβ)₂-type receptor tyrosine kinase family, which also includes the insulin-like growth factor 1 receptor and the insulin receptor–related receptor (IRR), and it plays a crucial role in regulating cell metabolism.^118–120^ Each IR αβ heterodimer consists of an extracellular ligand-binding α subunit and a transmembrane β subunit that contains the cytosolic tyrosine kinase domain.^119,121,122^ Insulin binding triggers a conformational change in IR, which activates its kinase activity and triggers autophosphorylation.^123^ This phosphorylation creates docking sites for insulin receptor substrate (IRS) proteins, including IRS-1 and IRS-2, which are then phosphorylated by the activated receptor.^124^

#### IGF and IGF receptor

Insulin-like Growth Factor (IGF) refers to a group of polypeptide hormones produced by the liver and other tissues, with a structure and function similar to insulin. There are two types, IGF-1 and IGF-2, which are crucial for cell proliferation, differentiation, and growth, and are involved in growth hormone (GH) regulation.^125^ IGFs exert their effects by binding to IGF receptors (IGFR), which include two main types: IGF-1 receptor (IGF-1R) and IGF-2 receptor (IGF-2R). IGF-1R is a transmembrane tyrosine kinase receptor consisting of two α subunits and two β subunits.^126^ The α subunit binds IGF-1, while the β subunit has tyrosine kinase activity.^127^ While both IGF-1R and IR play roles in cell proliferation, differentiation, survival, and metabolic regulation, IGF-1R is more focused on promoting cell growth and differentiation, whereas IR is more critical for glucose metabolism.^120^ In contrast, IGF-2R, also known as the mannose-6-phosphate receptor, lacks tyrosine kinase activity and regulates IGF-2 by binding and internalizing it for degradation in lysosomes.^127,128^

When IGF binds to IGF-1R and insulin binds to IR, two major signaling pathways are activated: the PI3K/Akt pathway and the Ras/MAPK/Erk pathway.^129^ The IRS-initiated PI3K/Akt pathway primarily regulates metabolic processes, while the SHC-initiated Ras/MAPK pathway controls cell growth and differentiation. These pathways involve complex interactions among various signaling molecules and proteins, working together to achieve specific biological outcomes.

#### Insulin/IGF signaling in glucose metabolism

The activated PI3K/Akt signaling pathway plays a crucial role in regulating glucose metabolism. Akt phosphorylates and inhibits GSK-3, which increases glycogen synthesis by reducing the inhibition of glycogen synthase, the key enzyme responsible for converting glucose to glycogen in the liver and muscle. When GSK-3 activity is high, glycogen synthesis decreases, leading to higher blood glucose levels.^130^ Besides its direct effect on glycogen synthesis, GSK-3 may also influence gluconeogenesis and glycogenolysis by regulating key enzymes including glucose-6-phosphatase and phosphoenolpyruvate carboxykinase.^131^ Further research on the regulation of GSK-3 across different tissues and conditions is essential for developing new therapeutic strategies for metabolic disorders such as diabetes and obesity.

Akt facilitates the movement of GLUT4 to the cell membrane, thereby increasing glucose uptake in muscle and adipose tissues.^132^ In its basal state, GLUT4 is stored in vesicles within the cell, and upon insulin binding to IR, the number of GLUT4-containing vesicles that move to the cell membrane increases, promoting glucose uptake.^132,133^ Akt phosphorylates AS160, a protein that regulates Rab GTPase, which in turn enhances the movement of these vesicles toward the plasma membrane.^134^ Once GLUT4 is incorporated into the membrane, it significantly increases the cell’s capacity to take up glucose. While the core mechanisms of GLUT4 trafficking are understood, further research is required to fully elucidate the complex signaling pathways involved and to develop strategies that improve GLUT4 translocation for better management of metabolic disorders such as diabetes.

In addition to regulating glucose metabolism in normal cells, insulin/IGF also affects cancer cell metabolism by activating the Ras/MAPK/Erk pathway. In cancer cells, Erk phosphorylates PKM2 (pyruvate kinase M2), an enzyme crucial for glycolysis and metabolic reprogramming.^135^ Before phosphorylation, PKM2 exists primarily in a less active dimeric form, allowing glycolytic intermediates to be diverted into biosynthetic pathways that support rapid cell growth. Upon phosphorylation by Erk, PKM2 shifts to a more active tetrameric form, enhancing its ability to catalyze the conversion of phosphoenolpyruvate (PEP) to pyruvate and increasing glycolytic flux.^136^ This shift is essential for sustaining the high glucose metabolism and lactate production characteristic of the Warburg effect in cancer cells, thereby promoting cancer cell survival and proliferation.^135^ Erk-mediated phosphorylation of PKM2 plays a pivotal role in reprogramming cellular metabolism to meet the needs of rapidly growing cancer cells, underscoring its importance in cancer progression.

#### Insulin/IGF signaling in cell growth and proliferation

By binding to its receptors, insulin/IGF also functions as a growth factor through the PI3K/Akt/mTOR pathway. mTOR, a dual-specificity protein kinase, plays a central role in regulating anabolic processes by responding to nutrient availability and growth factors.^137^ Mechanistic Target of Rapamycin Complex 1 (mTORC1), formed with components like raptor and LST8, primarily regulates protein synthesis and cell growth, while mTORC2, involving components like rictor and mSin1, is involved in actin cytoskeleton regulation and cell survival.^138^ mTOR catalyzes the phosphorylation of targets such as S6K1 and 4E-BP1, promoting protein synthesis and cell growth.^139^ Akt indirectly activates mTORC1 by inhibiting the TSC1/TSC2 complex, a negative regulator of mTORC1.^140^ Despite advances in understanding mTOR signaling, the complete network of downstream effectors and their roles in protein synthesis, cell growth, and metabolism remains to be fully elucidated.

In addition to its effects on metabolism, insulin regulates key cell cycle proteins, such as cyclin D1, p21, and p27, to control cell growth. Akt increases the stability and translation of cyclin D1, driving cell cycle progression from the G1 to the S phase, thereby promoting cell proliferation.^141^ Mechanistically, Akt phosphorylates and inhibits Glycogen Synthase Kinase 3 Beta (GSK-3β), leading to increased transcription of cyclin D1 by stabilizing c-Myc and β-catenin.^142^ Akt also inhibits proteins involved in cyclin D1 degradation, enhancing its stability and promoting nuclear translocation.^143^

p21 (Cip1) and p27 (Kip1) are crucial cyclin-dependent kinase inhibitors that regulate the cell cycle by inhibiting progression at the G1/S and G2/M transition points.^144,145^ Akt phosphorylates p21 and p27, causing their translocation from the nucleus to the cytoplasm.^146^ In the cytoplasm, phosphorylated p21, and p27 cannot effectively inhibit CDK-cyclin complexes, leading to continued cell cycle progression and increased cell proliferation.^147^

Insulin/IGF signaling can promote cell proliferation by regulating gene transcription. Elk-1, a member of the ETS-domain transcription factor family, plays a key role in this process. Elk-1 has three conserved domains, including an ETS domain for DNA binding and a C-terminal region with MAPK phosphorylation sites.^148^ Upon activation by Erk, Elk-1 undergoes phosphorylation, leading to the loss of SUMO modification, which converts Elk-1 from a repressive to an active transcriptional form.^149^ Elk-1 regulates the expression of genes involved in cell growth and proliferation, such as Egr-1, c-Fos, c-Myc, and c-Jun, which are crucial for cell cycle progression and differentiation^150–152^ These transcription factors, particularly c-Fos and c-Jun, form the AP-1 complex, which activates genes responsible for various cellular processes, including proliferation, differentiation, apoptosis, and stress response.^153–156^

#### Insulin/IGF signaling in cell survival and apoptosis

Insulin/IGF signaling regulates cell survival and apoptosis through the PI3K/Akt pathway by phosphorylating BAD (BCL-2-associated death promoter).^157^ The BCL-2 family includes proteins that either support cell survival, such as BCL-2 and BCL-XL or promote cell death, such as BAD and BAX.^158^ The balance between these proteins, forming homodimers or heterodimers, determines cell fate. BAD promotes apoptosis by binding to and inhibiting anti-apoptotic proteins like BCL-2 and BCL-XL.^159^ However, when Akt phosphorylates BAD, it prevents this binding, thereby promoting cell survival. Further research is needed to fully understand the downstream effectors and signaling pathways modulated by phosphorylated BAD, especially in different cell types and disease contexts.

Forkhead box (FOXO) transcription factors, named after the Drosophila forkhead gene, are part of a large family with 19 subclasses, ranging from FOXA to FOXS.^160^ FOXO proteins regulate the transcription of target genes involved in processes such as cellular energy production, oxidative stress resistance, and cell viability and proliferation.^161^ The movement of FOXO transcription factors between the nucleus and cytoplasm is regulated by nuclear export signals (such as 14-3-3 proteins) and nuclear localization signals.^162^ FOXO protein activity and subcellular localization are often regulated by post-translational modifications such as phosphorylation, acetylation, and ubiquitination.^163^ Akt phosphorylates FOXO transcription factors, causing them to be sequestered in the cytoplasm by binding to 14-3-3 proteins, which prevents their translocation to the nucleus.^162^ Through this mechanism, Akt indirectly promotes cell survival. However, the specific mechanisms regulating FOXO localization and activity, particularly the role of post-translational modifications, require further investigation.

#### Insulin/IGF signaling in cell motility

Insulin/IGF signaling also plays a role in cell migration. Microtubules, key elements of the eukaryotic cytoskeleton, are made up of conserved α/β-tubulin heterodimers and play a vital role in processes like cell division, movement, and intracellular transport.^164^ To perform these functions, microtubules form specific arrays, which are regulated by microtubule-associated proteins (MAPs).^165^ Erk phosphorylates MAPs, altering microtubule stability and dynamics, which are important for cell division and transport.^166^

Focal adhesion kinase (FAK) is a non-receptor tyrosine kinase that is vital for cell migration and focal adhesion.^167,168^ FAK consists of several important domains, such as the focal adhesion targeting domain, the Four-point-one, Ezrin, Radixin, Moesin (FERM) domain, and the kinase domain.^169^ Activated FAK regulates cell-matrix adhesion by connecting the extracellular matrix to the actin cytoskeleton via integrin receptors, promoting cell movement through interactions with other proteins like paxillin and vinculin.^170,171^

Upon activation, FAK autophosphorylates at tyr397, creating a high-affinity binding site that attracts Src family kinases and other signaling molecules, triggering downstream pathways related to cell survival, proliferation, and migration.^172^ Erk phosphorylates FAK, contributing to cell motility and survival by modulating focal adhesion dynamics and integrin signaling. Erk also activates RSK, a serine/threonine kinase crucial for cell survival, growth, and proliferation.^173^ RSK phosphorylates substrates such as the transcription factor CREB and the apoptotic regulator BAD.^174,175^ Phosphorylation of CREB by RSK enhances gene expression related to cell growth and survival, while phosphorylation of BAD prevents it from inhibiting anti-apoptotic proteins like BCL-2, thereby promoting cell survival. Additionally, Erk signaling can lead to the transcriptional activation and stabilization of cyclin D1, promoting the transition from the G1 to the S phase of the cell cycle.

In summary, insulin/IGF signaling is a complex and finely tuned process that integrates multiple pathways to regulate various cellular functions, ensuring metabolic homeostasis, growth, and survival. Dysregulation of this signaling can lead to metabolic disorders such as diabetes, obesity, and insulin resistance syndromes.^176^ Understanding the details of insulin/IGF signaling pathways and their interactions is crucial for developing effective therapeutic interventions for these conditions.

### Glucagon signaling

#### Glucagon and glucagon receptor

Glucagon, a polypeptide hormone secreted by pancreatic α cells, is essential for regulating blood glucose levels. Its synthesis and secretion are primarily influenced by blood glucose levels,^177^ insulin levels,^178^ and neuromodulation.^179,180^ When blood glucose levels drop or during a state of hunger, glucagon secretion increases, promoting liver glycogen breakdown and gluconeogenesis, thereby raising blood glucose levels.^181,182^ α cells respond to changes in blood glucose by integrating signals from ion channels,^183,184^ paracrine factors,^185^ and nervous system regulation.^179,186^ These processes allow for rapid and effective adjustment of glucagon secretion, maintaining glucose homeostasis. The glucagon receptor (GCGR) is a class B G protein-coupled receptor (GPCR), crucial for glucose metabolism and homeostasis.^187^ GCGR consists of an extracellular ligand-binding domain and a transmembrane domain with seven helices, typical of GPCRs.^188^ Mutations in GCGR that affect its conformation or ligand binding can significantly impair signaling, leading to glucose homeostasis disorders.^189,190^

#### Glucagon signaling in glucose metabolism

The extracellular domain of GCGR binds glucagon, triggering a conformational change that activates the receptor. This activation leads to the coupling of GCGR to G proteins, primarily Gs, which stimulates adenylate cyclase activity and increases intracellular cyclic AMP (cAMP) levels.^191–193^ The elevation of cAMP triggers the activation of protein kinase A (PKA), which phosphorylates several downstream proteins, including CREB, leading to the transcriptional activation of glucose 6-phosphatase and phosphoenolpyruvate carboxykinase (PEPCK). This process enhances gluconeogenesis and glycogenolysis in liver cells.^194^

In addition to phosphorylating CREB,^195^ PKA also triggers multiple intracellular events. It phosphorylates and regulates phosphofructokinase 2 (PFK-2) and fructose 2,6-bisphosphatase (FBPase2),^196^ inhibiting PFK-2 activity and activating FBPase2, which leads to increased levels of fructose 6-phosphate, promoting gluconeogenesis and reducing glycolysis. PKA also stimulates pyruvate kinase, modulating levels of fructose 1,6-bisphosphate and pyruvate, leading to the suppression of glycolysis.^197^ Furthermore, PKA activates phosphorylase kinase, which facilitates the breakdown of glycogen into glucose 1-phosphate, while simultaneously inhibiting glycogen synthase.^198^

These mechanisms underscore the pivotal role of GCGR and PKA in regulating glucose homeostasis, promoting glucose production and release during periods of low blood glucose. This pathway illustrates how glucagon is translated into metabolic responses through intricate biochemical processes.

#### Glucagon signaling in amino acid metabolism

In the decades following its discovery, glucagon was typically regarded as a counter-regulatory hormone to insulin, with both hormones working together to regulate glucose levels and maintain homeostasis.^185^ Beyond its role in glucose homeostasis, glucagon binding to GCGR also regulates amino acid metabolism by increasing the activity of enzymes in the urea cycle at the transcriptional level.^199,200^ The capacity for ureagenesis largely depends on enzyme activity, with long-term regulation requiring the synthesis of five key enzymes.^201^ In rat hepatocytes, glucagon stimulates the amino acid transport system A, increasing amino acid uptake and providing substrates for ureagenesis.^202^

Glucagon induces ureagenesis by increasing substrate availability through amino acid uptake and by upregulating the transcription of key enzymes like N-acetyl glutamate synthase (NAGS).^201^ NAGS converts acetyl-CoA and glutamate to N-acetyl-glutamate (NAG), the essential activator of carbamoyl phosphate synthetase-1 (CPS-1), one of the enzymes initiating ureagenesis.^203^ This dual regulation allows for both long-term and rapid activation of ureagenesis.^204^ Furthermore, glucagon may activate other metabolic products that influence NAGS activity. Blockade of the glucagon receptor, either through GCGR gene knockdown or antagonists, leads to increased plasma amino acid levels and a decrease in ureagenesis.^205,206^ Blocking glucagon receptor signaling decreases the expression of genes responsible for hepatic amino acid uptake, resulting in elevated amino acid levels.^205,207^ However, other mechanisms by which glucagon influences the key enzymes in ureagenesis remain to be fully understood.

#### Glucagon signaling in lipid metabolism

The glucagon receptor is recognized as a potential target for hypolipidemic therapies due to its role in modulating lipid metabolism. Glucagon can influence the expression and activity of peroxisome proliferator-activated receptors (PPARs), a group of ligand-activated nuclear receptors that function as transcription factors.^208^ There are three isoforms of PPARs: PPAR-α, PPAR-β/δ, and PPAR-γ, with PPAR-α and PPAR-γ primarily involved in regulating lipid metabolism, insulin sensitivity, and glucose homeostasis.^209^ Glucagon stimulates PPAR-α, promoting fatty acid β-oxidation, reducing triglyceride levels, and increasing HDL cholesterol.^210^ Glucagon also influences PPAR-γ, a key regulator of adipocyte differentiation and insulin sensitivity.^211^ This interplay between glucagon and PPARs underscores the intricate regulation of lipid metabolism and storage, highlighting their importance in the body’s metabolic adaptation to physiological changes.

In hepatocytes, glucagon influences lipid metabolism through a detailed intracellular mechanism. Upon binding to its receptor, glucagon activates cAMP and CREB, leading to increased expression of carnitine palmitoyltransferase 1 (CPT-1).^212^ CPT-1 is essential for lipid metabolism, converting long-chain fatty acids into acyl-carnitine for mitochondrial β-oxidation.^213^ Additionally, glucagon activates PKA, which inhibits acetyl-CoA carboxylase, reducing malonyl-CoA levels and relieving inhibition of CPT-1. This process promotes β-oxidation and decreases fatty acid synthesis, preventing the re-esterification of free fatty acids (FFAs) to triglycerides and the release of very low-density lipoproteins (VLDL). This mechanism allows cells to utilize stored fatty acids for energy, particularly when glucose is scarce, enhancing lipid metabolism. However, further research is needed to explore other potential interactions within glucagon signaling.

Glucagon plays a crucial and diverse role in regulating metabolic processes. By regulating blood glucose levels, glucagon ensures a steady energy supply, especially during fasting or low carbohydrate intake. Its role in amino acid metabolism through ureagenesis underscores its importance in nitrogen waste management and maintaining amino acid balance. In lipid metabolism, glucagon’s activation of PPARs and CPT-1 supports the utilization of stored fats for energy, contributing to metabolic flexibility. This comprehensive regulation by glucagon highlights its critical function in maintaining metabolic homeostasis, making it a key target for therapeutic strategies in metabolic disorders such as diabetes, hyperlipidemia, and obesity.

### Somatostatin signaling

#### Somatostatin and somatostatin receptor

Somatostatin was first isolated from pigs and found to inhibit the secretion of pituitary growth hormone in both rats and humans.^214^ Initially believed to be a product of hypothalamic neurons, somatostatin was later found to be secreted by the δ cell.^13,215^ It was soon discovered that somatostatin inhibits the secretion of insulin and glucagon from the pancreas, as well as gastrin from the stomach.^13,185,216,217^ Somatostatin exerts its effects through a family of G-protein-coupled receptors known as somatostatin receptors (SSTRs), which include five subtypes (SSTR1-5), each with distinct tissue distributions and functions.^218,219^

#### Somatostatin signaling in the regulation of hormones

Somatostatin receptors (SSTRs) are widely distributed in various tissues, including the pancreas, where they mediate somatostatin’s inhibitory effects on both endocrine and exocrine functions.^220–224^ In the central nervous system (CNS), somatostatin suppresses the secretion of growth hormone (GH), prolactin (PRL), thyroid-stimulating hormone (TSH), and adrenocorticotropic hormone (ACTH) from the anterior pituitary.^214,225–227^ In the pancreas, somatostatin is secreted in response to elevated blood glucose levels and certain amino acids, inhibiting the release of hormones such as insulin and glucagon.^228^ It also suppresses the secretion of various gastrointestinal hormones, including cholecystokinin (CCK), gastrin, secretin, vasoactive intestinal peptide, motilin, and gastric inhibitory polypeptide.^229^ In terms of exocrine function, somatostatin inhibits the secretion of gastric acid, bicarbonate, and digestive enzymes, ensuring a balanced hormonal environment and preventing excessive hormone activity that could disrupt metabolic homeostasis.^228,230,231^

Somatostatin exerts its effects by binding to SSTRs, which inhibit adenylate cyclase activity, reduce intracellular cAMP levels, and modulate ion channel activity.^219,232^ This modulation affects Ca^2+^ levels, directly influencing the endocytosis and exocytosis of hormones.^233^ Additionally, somatostatin can influence Ca^2+^ channels through a cGMP-dependent protein kinase pathway.^234^

In pancreatic α and β cells, somatostatin binds to SSTR2 and SSTR5 receptors, activating the inhibitory G (Gi) protein, which inhibits adenylate cyclase.^235^ This inhibition reduces intracellular cAMP levels, leading to decreased PKA activity and ultimately inhibiting glucagon and insulin secretion. Reduced PKA activity inhibits voltage-dependent calcium channels (VDCCs), which are essential for Ca^2+^ influx—a critical step for hormone granule mobilization and fusion.^236,237^ Without sufficient Ca^2+^ influx, the exocytosis of hormone-containing granules is impaired, reducing hormone secretion. Additionally, decreased PKA activity may reduce the phosphorylation of transcription factors such as CREB, which typically promotes the transcription of genes necessary for glucagon and insulin synthesis, further decreasing hormone secretion.^238^

#### Somatostatin signaling in antiproliferation

Somatostatin plays an essential role in antitumor effects through various direct and indirect mechanisms.^239^ At the mechanistic level, somatostatin receptor signaling, especially via SSTR1 and SSTR5, interferes with growth factor receptor pathways by diminishing the phosphorylation of key proteins like EGFR, MAPKs, and components of the PI3K/Akt pathway—critical regulators of cell survival.^240^ Activation of SSTR5 also leads to the dissociation of EGFR/ErbB2 heterodimers, which are important for receptor tyrosine kinase autophosphorylation and the initiation of downstream signaling.^241^ Protein tyrosine phosphatases (PTPs) also contribute to this mechanism by removing phosphate groups from tyrosine kinases associated with growth receptors.^242^ PTPs, specifically Src Homology Region 2 Domain-Containing Phosphatase-1 (SHP-1), Src Homology Region 2 Domain-Containing Phosphatase-2 (SHP-2), and DEP-1/PTPeta, are recognized as downstream effectors of SSTRs, transmitting antiproliferative signals.^243^ Somatostatin blocks the cell cycle through PTPs, with SHP-1 regulating cell-cycle components such as CDK2, p27, and cyclin D1.^244^

In addition, somatostatin receptors trigger apoptosis, with SHP-1 being essential for the cytotoxic signaling that causes intracellular acidification and ultimately cell death.^245,246^ SSTR1, SSTR3, and SSTR4 suppress the Na^+^-H^+^ exchanger (NHE1), resulting in intracellular acidification, which may contribute to somatostatin’s resistance to cell migration in certain tumor types.^247,248^ Through these mechanisms, somatostatin binds to SSTRs, functioning as an antiproliferative agent and a potential target for various tumor types.

### Pancreatic peptide signaling

#### Pancreatic peptide and its receptor

Pancreatic peptide (PP) is a member of the neuropeptide Y (NPY) family, secreted by the PP cell and primarily distributed in the pancreas.^249,250^ X-ray crystallography has revealed the structure of PP, characterized by a typical ‘PP-fold.’ This fold consists of a polyproline helix (residues 2–8) and an α-helix (residues 14–32), linked by a β-turn, creating a hairpin-like formation with a small hydrophobic pocket.^251^ This structure is crucial for its binding to neuropeptide receptors (NPYRs), a group of Gi-protein-coupled receptors (GPCRs) that inhibit cAMP production.^252,253^ Four subtypes of NPYRs (Y1, Y2, Y4, and Y5) have been identified in humans, with PP acting as a selective agonist for Y4.^254^

Functionally, PP has been found to inhibit appetite and islet cell function.^255,256^ Y4, in particular, is known to suppress appetite and reduce body weight, whereas Y1 and Y5 have the opposite effect.^257^ Y4 also influences islet function, including β-cell mass and the release of glucagon and somatostatin.^258,259^ Beyond its role in the pancreas, PP is involved in the gut-brain axis, collaborating with other hormones in the brain and pancreas to maintain overall metabolic homeostasis.^260^ Thus, the main physiological effects of PP include inhibiting gastric emptying and appetite while enhancing energy expenditure.^261^

#### Pancreatic peptide signaling

However, pancreatic peptide (PP) signaling has not been extensively studied, and there is limited research on its mechanisms and effects. In pancreatic β cells, when PP binds to its Gi protein-coupled receptor on the membrane, it decreases cAMP levels, leading to the inhibition of PKA and subsequent actions mediated by PKA, including the secretion of glucagon and insulin.^262^ Further investigation is needed to uncover additional signaling pathways and downstream effectors of PP, to better understand its role in hormone regulation.

## Pancreatic exocrine signaling

### Pancreatic exocrine components

The pancreatic exocrine part consists of acinar cells and ductal cells.^263^ Pancreatic exocrine function is crucial for the gastrointestinal tract as it secretes digestive enzymes and bicarbonate ions into the duodenum, aiding in food digestion.^264^ This complex process is regulated by a network of hormonal and neural signals that ensure proper enzyme secretion in response to food intake.^265^

Acinar cells, which make up nearly 90% of the pancreas, are essential for producing digestive enzymes. These enzymes include amylase for carbohydrate digestion, lipase for breaking down fats, and proteases, which are crucial for protein digestion.^266^ Ductal cells, on the other hand, transport these enzymes and secrete bicarbonate, which, along with digestive enzymes, forms pancreatic juice. This juice neutralizes gastric acid and provides the appropriate pH in the duodenum for digestion.^267,268^

#### Exocrine signaling in secretion

Cytoplasmic Ca^2+^ is vital for the release of digestive enzymes in pancreatic acinar cells.^269^ Stimulants such as acetylcholine and cholecystokinin (CCK) bind to their respective receptors, activating Gq or Gi proteins, which in turn activate phospholipase C (PLC). PLC breaks down phosphatidylinositol-4,5-bisphosphate (PIP2) into inositol triphosphate (IP3) and diacylglycerol (DAG).^270^ IP3 binds to receptors on the endoplasmic reticulum, triggering Ca^2+^ release into the cytoplasm from the endoplasmic reticulum.^269^ The increased cytosolic Ca^2+^ concentration activates various Ca^2+^-dependent proteases, promoting the secretion of pancreatic enzymes and fluid.^271–273^

The cAMP/PKA pathway also plays a role in pancreatic secretion. The secretin receptor, a class B GPCR, activates the Gs protein upon binding with secretin, leading to the activation of cAMP and subsequent activation of PKA.^274^ PKA phosphorylates several critical enzymes and ion channel proteins, such as CFTR, which facilitates Cl⁻ secretion and regulates pancreatic secretion.^275^

**Fig. 4:**
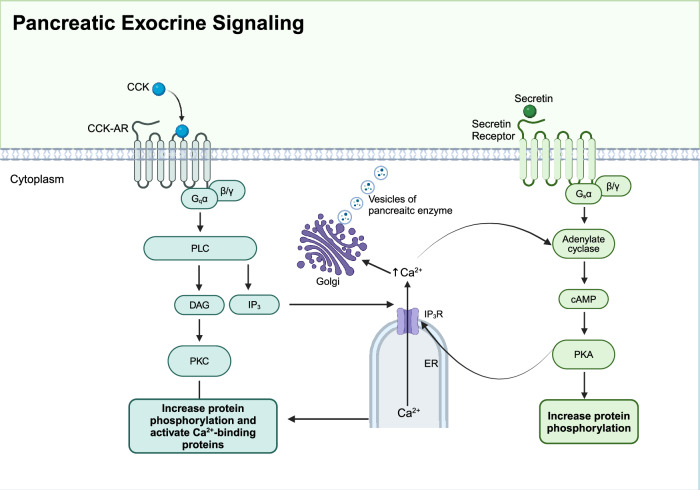
Pancreatic exocrine signaling. Two signaling pathways are involved in pancreatic exocrine function. Upon stimulation by acetylcholine or cholecystokinin (CCK), Gq protein-coupled receptors activate phospholipase C (PLC), which hydrolyzes phosphatidylinositol 4,5-bisphosphate (PIP2) into inositol triphosphate (IP3) and diacylglycerol (DAG). IP3 binds to its receptors (IP3R) on the endoplasmic reticulum (ER), triggering Ca^2+^ release into the cytoplasm. Increased intracellular Ca^2+^ concentration activates protein kinase C (PKC) and Ca^2+^-binding proteins, promoting enzyme secretion. Secretin binding to its receptor activates adenylate cyclase via Gs protein, increasing cAMP levels and activating protein kinase A (PKA), which also enhances enzyme secretion. This coordinated signaling ensures the efficient digestion of nutrients. Created in BioRender.com

Ca^2+^ and cAMP/PKA signaling pathways often interact and cooperate in regulating pancreatic exocrine function. Ca^2+^ can enhance cAMP production through Ca^2+^-dependent phospholipase, while PKA can regulate intracellular Ca^2+^ concentrations by phosphorylating certain Ca^2+^ channels or pumps (Fig 4).^276^ This cross-regulatory mechanism ensures precise control of pancreatic exocrine secretion, with both pathways playing key roles in the efficient secretion of digestive enzymes and electrolytes.

### Regulation of exocrine function

#### Hormonal regulation

When food enters the duodenum, cholecystokinin (CCK) secreted by the small intestine stimulates acinar cells to secrete digestive enzymes and fluid. Initially, the role of CCK in directly stimulating acinar cells was questioned because humans lack high-affinity CCK-A receptors.^277,278^ It was suggested that CCK acts on vagal afferent fibers, indirectly mediating acinar cell secretion through neurotransmitters.^265^ However, in 2008, scientists discovered that CCK can bind to receptors on the membrane, activating intracellular Ca^2+^ and subsequent Ca^2+^-dependent exocytosis in acinar cells.^279^

When acidic chyme from the stomach enters the duodenum, S cells in the duodenum secrete secretin, which is crucial for bicarbonate secretion.^280^ Secretin acts on ductal cells, stimulating fluid and bicarbonate secretion by increasing cAMP levels.^281^ The pancreatic fluid, rich in various digestive enzymes secreted by acinar cells, contains a small amount of NaCl. As Cl⁻ flows through the interlobular ducts, it is absorbed by ductal cells, which then secrete bicarbonate and water into the pancreatic ducts.^282^ Additionally, CCK also acts on ductal cells, enhancing fluid secretion by potentiating the effects of secretin.^283^

#### Neural regulation

Pancreatic secretion is regulated by neural pathways, including both the gut-brain axis and the intrapancreatic system. The intrapancreatic network receives inputs from preganglionic parasympathetic (vagal) fibers, postganglionic sympathetic (splanchnic) fibers, and potentially other fibers stemming from the gut wall. Various neurotransmitters are involved in the neural regulation of exocrine function.^282^ Acetylcholine, released by parasympathetic nerves, acts on pancreatic acinar and ductal cells to increase intracellular Ca^2+^ concentration, promoting the secretion of enzymes and fluids.^284^ Vasoactive intestinal peptide (VIP) and adenosine triphosphate (ATP), also from parasympathetic nerves, stimulate pancreatic fluid secretion.^285,286^ Neuropeptide Y (NPY) regulates blood flow and inhibits pancreatic secretion. Additionally, substance P and calcitonin gene-related peptide (CGRP) act as inhibitory agents, reducing pancreatic secretion.^287^

## Endocrine-exocrine crosstalk

It was previously believed that the endocrine and exocrine parts of the pancreas are structurally and functionally independent of each other. However, accumulating evidence has indicated that the endocrine and exocrine pancreas have close communication and interaction. And crosstalk between the two parts may play a critical role in the pathogenesis of diseases. “Endocrine-exocrine crosstalk” can be defined as the interaction of the two components through paracrine signals or alteration in extracellular contexts. For example, the exocrine pancreas can influence the physical properties and function of the endocrine pancreas, and vice versa.

### Anatomic foundation for endocrine-exocrine crosstalk

**Fig. 5:**
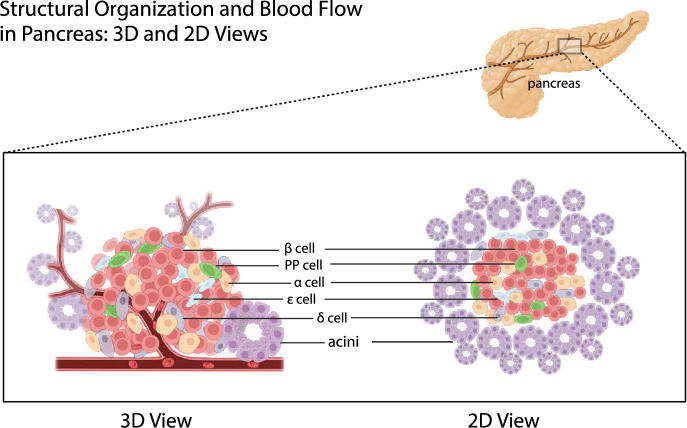
Structural organization and blood flow in the pancreas: 3D and 2D views. The pancreas comprises various cell types, including acinar cells responsible for exocrine function and islet cells (α, β, δ, PP, and ε cells) responsible for endocrine function. Acinar cells are shown in clusters forming acini, while islet cells are scattered within the pancreatic tissue. The blood flow direction is indicated, starting from the branches of pancreaticoduodenal and splenic arteries entering the pancreas, first passing through the islets of Langerhans, and then flowing into the exocrine acinar and ductal cells. Created in BioRender.com

Anatomy of the pancreas in both humans and rodents revealed that arterial blood from the pancreas entered the pancreas via branches of pancreaticoduodenal arteries (upper and lower) and splenic arteries.^288,289^ These branches further divide into a network of arterioles and capillaries, first entering the islets of the Langerhans.^290^ Blood flows out of the capillaries in the islets and into the venules. The blood that flows from the islets continues into a larger network of veins and capillaries, which resupply the exocrine acinar cells and ductal cells of the pancreas^288,289^(Fig. 5).

This blood supply route from the islets to the exocrine part ensures that endocrine hormones (such as insulin and glucagon) can directly affect the function of exocrine cells and coordinate the overall function of the pancreas.

Handerson wrote in his essay that he observed all the endocrine organs and found that all these organs were organized by compact structure, only the endocrine pancreas islets were scattered in the pancreas, connecting closely with the exocrine pancreas in the physical distance.^291^ Since then, increasing activity has focused on understanding various aspects of this unusual anatomy. There are approximately 500 lobules within the rat exocrine pancreas. And there are about 400 islets, equally distributed within the lobules or between the tissue spaces along the secretory ducts.^288^

The exocrine pancreas can be divided into two regions, juxta- and tele-insular regions. In tele-insular regions away from the islets, the acinar cells and their nucleus are smaller than the juxta-insular regions which are in immediate proximity.^292^ Since previous studies have found that insulin promotes secretion and growth of acinar cells,^293,294^ it is reasonable to assume that the blood vessels of the pancreas may play a role in transporting hormones secreted by the islets to acinar cells in the juxta-region first (Fig. 5). The scattered distribution of islets in the exocrine part also makes people speculate that this phenomenon is caused by paracrine action. While the presence of paracrine signaling within the islets is acknowledged,^295^ whether paracrine signaling takes part in the crosstalk between endocrine and exocrine pancreas and the specific roles and underlying mechanisms remain unclear. Understanding how insulin and other hormones influence acinar and ductal cells through paracrine actions could reveal new therapeutic targets.

Recent studies have highlighted the complex interactions between acinar cells and pancreatic beta cells, revealing how disruptions in this communication can impact β cell function. For instance, excess pancreatic elastase, an enzyme produced by acinar cells, has been shown to impair acinar-beta cell communication by disrupting mechano-signaling pathways. This disruption can lead to altered insulin secretion and contribute to beta cell dysfunction, which is particularly relevant in the context of pancreatitis and other pancreatic disorders.^296^

In healthy conditions, the specific tissue structure and distribution in the pancreas allow the endocrine and exocrine parts of the pancreas to work together to maintain the overall balance of hormonal and digestive functions in the body. However, under the influences of certain endocrine and exocrine pancreatic diseases, the pancreatic parenchyma including acini, ducts, and islets, as well as surrounding tissues such as blood vessels are destroyed, resulting in endocrine and exocrine dysfunction of the pancreas. Understanding the mechanisms behind this tissue destruction and dysfunction is crucial for developing therapeutic strategies aimed at preserving pancreatic function or mitigating damage. Additionally, exploring how early detection and intervention might prevent the progression of such diseases could lead to more effective treatments, potentially preserving both endocrine and exocrine functions before irreversible damage occurs.

### Epidemiology of diseases with endocrine-exocrine crosstalk

#### From exocrine diseases to diabetes

Epidemiological studies have shown that pancreatic exocrine diseases including PDAC, pancreatitis, and cystic fibrosis (CF), often co-exist with metabolic disorders, such as diabetes mellitus, highlighting the significance of the endocrine-exocrine crosstalk. These epidemiological studies provide data support for the communication between the endocrine and exocrine pancreas, from a perspective of pancreatic diseases.

PDAC is one of the ten most common cancers in the world, notorious for its dismal prognosis with a 5-year survival of 13%.^297^ PDAC is presently the third most common cause of cancer-related mortality and is expected to rise to the second leading cause of cancer deaths in the United States.^298^ Unluckily, a large number of PDAC patients lost opportunities for surgical intervention,^299^ due to its asymptomatic traits in the early stage and lack of specific detection methods.^300^

PDAC and diabetes are closely related, and longstanding T2D is a risk factor in the initiation of PDAC.^2^ However, new-onset diabetes is thought to be more associated with the development of PDAC,^3^ and is considered an early manifestation of PDAC. A prospective study showed that 74%–88% of PDAC patients with diabetes were diagnosed with diabetes within the last 24 months.^301,302^ And the diabetes subsided after pancreaticoduodenectomy in 57% of patients with new-onset diabetes. Another retrospective study noted that 50% of PDAC patients with diabetes were new-onset.^303^ This new-onset diabetes, named PDAC-associated DM (PDAC-DM), is one of the type 3c diabetes mellitus whose pathogenesis is unknown. Moreover, PDAC-DM is not distinguished from other types of diabetes by clinical manifestations and signs. Early detection of PDAC holds the most promise in terms of improving long-term outcomes, and understanding the pathogenesis of PDAC-DM could help in obtaining biomarkers for the early diagnosis of PDAC from the population with new-onset diabetes.^304^

Acute and chronic pancreatitis is the third leading cause of gastrointestinal-related hospitalization in the United States by 2021.^305^ Acute pancreatitis (AP) is caused by many factors. The clinical symptom usually manifests acute abdominal pain and high levels of serum amylase.^306^ AP leads to necrosis of pancreatic parenchyma pathologically. And frequent AP onset can turn into chronic pancreatitis (CP) characterized by chronic progressive pancreatic inflammation and scarring with pancreatic parenchymal calcifications, ultimately leading to pancreatic exocrine and endocrine insufficiency.^307,308^

Individuals with CP face a higher risk of developing diabetes. A prospective cohort study tracking 500 CP patients over an average of 7 ± 6.8 years revealed that 25 years after CP onset, the cumulative incidence of diabetes ranged from 79% to 87%. Additionally, distal pancreatectomy was identified as an independent risk factor for the development of diabetes.^4^ In addition to that, a cohort study including 2011 patients with CP showed 564 patients developed diabetes during the follow-up period (median duration of 22.0 years). The cumulative incidence of DM at 20 and 50 years after the onset of CP was 45.8% (95% CI, 41.8%–50.0%) and 90.0% (95% CI, 75.4%–97.7%) respectively.^309^ It appears that the longer the duration of CP, the higher the prevalence of CP-related DM.

CF is an autosomal recessive disorder characterized by mutations in the cystic fibrosis transmembrane conductance regulator (CFTR) gene.^310^ Mutations in CFTR lead to altered sodium and chloride permeability on the cellular epidermis,^311,312^ and abnormal mucus secretion.^313^ In the pancreas, this manifests as blockage of the ducts by abnormal mucus secretion, limiting the release of digestive enzymes and leading to dyspepsia.

Cystic fibrosis-related diabetes mellitus (CFRD), a major complication of CF, is present in 2% of children, 19% of adolescents, and 40%–50% of adult CF patients.^314^ The development of diabetes is independently associated with CFTR.^315^ However, CFRD is not an autoimmune disease similar to T1D.^316^ The occurrence of macrovascular complications and consequent death is extremely low. However, CFRD is strictly correlated with decreased lung function in CF patients.^317^ And respiratory failure is the main cause of death in CFRD patients.

CFRD is not a type of autoimmune disease. But it is similar to T1D with insufficient insulin secretion, which can develop at a young age and is usually not associated with the presence of metabolic syndrome (hypertension, abdominal obesity, hyperlipidemia). Although CFRD is less common before puberty, Yi et al. found that abnormal glucose tolerance (AGT) was found in 39% of infants and children with CF between the ages of 3 months and 5 years.^318^ These children with AGT were at greater risk of progressing to CFRD. Even patients with CF who had normal glucose tolerance (NGT) were found to have deficient insulin secretion in the study.^319^ In a two-year follow-up of patients with CF, CF patients preferentially exhibit impaired first-phase insulin secretion, with insulin output showing a decreasing effect over time in both oral and intravenous glucose tolerance tests.^320,321^ The progression of CFRD can progress sequentially through glycemic uncertainty, impaired glucose tolerance, CFRD without high fasting glucose, and CFRD with high fasting glucose. Although these states may shift back and forth due to external factors such as infections, the overall trend is toward diabetes.^322^ A better understanding of the pathophysiology and pathogenesis of CFRD, and early prognosis of CFRD, play an important role in the decline of pulmonary function and improve survival.^314^ In recent years, there has been increasing clinical attention regarding the treatment of CFRD, but there are still many problems with the treatment. Since it is often misdiagnosed as T2D, it is extremely important to develop appropriate diagnostic criteria. Compared with T1D and T2D, the molecular mechanism of CFRD is still less studied, and its pathogenesis is still not elucidated, making the development of specific therapies very difficult.

#### From diabetes to PDAC

Diabetes is a group of diseases defined by persistent hyperglycemia. As of 2021, there are 529 million people diabetes patients worldwide, and the global age-standardized total diabetes prevalence was 6.1%.^323^ Type 1 diabetes (T1D), accounting for 5%–10% of the total diabetes cases worldwide,^37^ is characterized by absolute insulin deficiency. T2D, the most common form of diabetes, initially arises from reduced insulin sensitivity, followed by an insufficient compensatory insulin response.^324^ While T2D is widespread, diabetes can also result from other conditions, particularly those affecting the exocrine pancreas.^2^ Historically termed pancreatogenic diabetes mellitus, this form is now commonly referred to as type 3c diabetes. The primary causes include chronic pancreatitis (79%), pancreatic ductal adenocarcinoma (PDAC) (8%), hemochromatosis (7%), cystic fibrosis (CF) (4%), and prior pancreatic surgery (2%).^325^ The prevalence of type 3c diabetes can be reasonably estimated at 1%–9%.^326^ However, some research also indicates that new-onset diabetes may be a result of pancreatic exocrine diseases like PDAC as a paraneoplastic outcome.^3,327^

Diabetes is one of the risk factors for PDAC, and extensive research supports the notion that diabetes can increase the risk of PDAC.^328,329^ A meta-analysis involving 2192 PDAC patients and 5113 controls, revealed a correlation between diabetes and a 1.8-fold increased risk of PDAC [95% confidence interval (CI) = 1.5–2.1].^330^ The risk of PDAC is negatively correlated with the duration of diabetes.^331^ Patients diagnosed with diabetes for less than one year have a significantly higher risk of PDAC compared to other patients. A population-based cohort study indicated that among 2122 diabetes patients aged over 50 years old, 18 individuals (0.85%) were found to have PDAC within three years of diabetes diagnosis, representing an eight-fold increase compared to the expected PDAC incidence.^332^ Moreover, 10 cases (56%) were discovered to have PDAC within the first six months of the initial diabetes diagnosis. While the above-mentioned studies suggest a potential association between newly diagnosed diabetes and the onset of PDAC, the effectiveness of using new-onset diabetes as an early marker for PDAC still requires further research and exploration.

### Mechanisms of exocrine diseases causing diabetes

#### Destruction of pancreas parenchyma

PDAC, pancreatitis, and CF are all related to the destruction of pancreas parenchyma. Pancreaticoduodenectomy directly resulted in the loss of islet cells, although the remaining beta cells also appeared to be dysfunctional.^304,333^ The inflammation and fibrosis associated with CP cause a decrease in pancreatic volume and lead to the loss of β cells.^334^ In fact, there is also inflammation-induced β cell dedifferentiation in CP patients, which is one of the mechanisms of CP-induced diabetes.^335^ Interestingly, α cells seem to maintain a certain amount and volume in CP patients and may even increase.^336^ The increased glucagon levels in CP patients may be related to this and then promoted the development of CP-related DM.^337^ However, the reason of maintaining in α cell amount needs further investigation. As the pancreatitis progresses, it can lead to extensive fibrosis and fatty infiltration of the pancreatic parenchyma.^338^ In this process, pancreatic stellate cells (PSC) have taken a critical part, which is activated by the pancreatic damage, and then produce an extracellular matrix (ECM) in response to these damages.^339^ Acute pancreatitis-induced diabetes is caused by β cell damage and insufficient regeneration, and in mice with acute pancreatitis, Krt5-positive cells can transdifferentiate to form β cells, partially compensating for the loss.^106^ Interestingly, diabetes was also common in patients with mild pancreatitis without pancreatic necrosis.^340^ The reasons behind this are worth exploring. In pancreatitis without pancreatic necrosis, chronic inflammation is also likely to be present, and the release of cytokines may be associated with insulin resistance. Besides, in the absence of necrosis, pancreatitis may also lead to a stress response and dysfunction of islet cells. This damage is not sufficient to trigger necrosis but is sufficient to interfere with insulin secretion. Some other possible mechanisms include alterations in the pancreatic microenvironment, accumulation of metabolites due to inflammation, or a hyper-autoimmune response of the pancreas to mild pancreatitis.

In terms of CF, abnormal function of CFTR protein leads to abnormal chloride and bicarbonate concentrations in pancreatic tissues, accompanied by blockage of the pancreatic ducts and damage to the pancreatic epithelium.^341^ Persistent pancreatic ductal obstruction, inflammation, fibrosis, and fatty infiltration lead to the destruction of the pancreatic parenchyma.^342^ Immunohistochemical analysis of pancreatic islets in patients with CFRD has shown a marked reduction in both islet volume and β cell numbers compared to those without CFRD.^343,344^ Glucagon secretion was also suppressed in CFRD patients with impaired exocrine secretion.^345^ The damage to β cells in CFRD patients appears to be non-selective, with the exocrine function also being compromised due to ongoing inflammation, fibrosis, and fatty infiltration.^346^ β cell deficiency in children may lead to impaired glucose tolerance and the later development of CFRD.^347^ In addition to this, there are a number of possible factors that can affect the preserved islet, and the remaining islet does not appear to function well for insulin secretion due to distorted blood flow with the rest of the body.^322,348^ Although diabetes usually develops in advanced stages of CP,^349^ β cell dysfunction and apoptosis have been found to precede the onset of DM,^350^ which leads to speculation that the dysfunction of β cells is one of the essential mechanisms in the pathogenesis of CP-related DM.

#### Inflammatory infiltration

The immune response within the pancreas, particularly the inflammatory environment surrounding islet cells, has a direct impact on islet cell function. Chronic inflammation and excessive activation of the immune system can lead to islet cell dysfunction and apoptosis.^351^ This is particularly evident in T1D, where the immune system’s attack on β cells is a major cause of insulin deficiency.^352^

PDAC is a highly aggressive tumor with inflammatory infiltration of the pancreas. Inflammatory factors constitute a chronic inflammatory microenvironment that directly contributes to the impairment of β cells.^353^ Macrophage migration inhibitory factor (MIF) expression is upregulated in PDAC tissues, resulting in impaired insulin secretion from β cells. Serum MIF levels were markedly elevated in PDAC patients with new-onset diabetes compared to controls, indicating that MIF could serve as a potential biomarker for early PDAC detection.^354^ In addition to this, acute inflammation may also be involved in the development of PDAC-DM.

Gao et al. observed significantly elevated serum levels of the inflammation marker CRP and the inflammatory mediator TNFSF13 in patients with PDAC-DM, which notably decreased after lesion resection.^355^ This finding suggests that acute inflammation could be a potential target for diagnosing and treating PDAC-DM.

In terms of pancreatitis, one of the early events in pancreatic necroinflammation is known to be the upregulation of pro-inflammatory cytokines.^356^ On the one hand, high expression of the IL-1β and IL-1R are able to directly induce β-cell apoptosis,^357^ resulting in a reduction in β-cell number. Antagonists of IL-1β and IL-1R were confirmed efficient against T2D in clinical trials.^358^ On the other hand, changes in the internal environment of the pancreas in CP caused by chronic inflammation lead to disordered cellular crosstalk and signaling mechanisms, resulting in altered cellular function.^359^ Perforin enables CD8^+^T cell priming β cell apoptotic program, whereas CD4^+^T cell-mediated β cell destruction depends mainly on Fas/FasL, IFN-γ and TNF-α. In addition, IL-1β can also promote insulin secretion. However, in the inflammatory environment of CP, IL-1β can persistently interact with IFN-γ and TNF-α, which induces persistent β cell destruction by CD4^+^T cells.^360^ Th1 cells were found to be more abundant in circulation and islets of CP diabetic patients than those without diabetes and controls. It may be attributed to the immune dysregulation caused by chronic inflammation environment. Specifically, Th1 cells are able to secrete the IFN-γ, which is capable of reducing the nuclear localization of Pdx1.^361^ Transcription factors Pdx1 plays an essential role in the β cell development and maturation, and Pdx1 can combine with regulatory elements to regulate insulin gene expression.^362^ And the reduction of Pdx1 was confirmed to be related to β cell dysfunction.^363^ However the molecular mechanisms underlying this remain to be investigated. Adrenomedullin and vanin-1 are carried by exosomes in many inflammatory responses, possibly taking part in β cell dysfunction in CP-related DM and PDAC-DM.^364–366^ Pro-inflammatory cytokines such as IL-10 and IL-12 need more studies to confirm their functions in β cell dysfunction to better distinguish CP-related DM from T1D and T2D. CP and DM are both known risk factors for PDAC. The persistent inflammation and fibrosis of the pancreas caused by CP not only destroy the function of islet cells, leading to DM but also increase the risk of cancer by changing the microenvironment.^356^ DM is considered to be one of the early manifestations of PDAC.^3,327^ Research indicates that the chronic inflammation seen in CP patients contributes to insulin resistance and β cell dysfunction, raising the risk of diabetes. In turn, diabetes fosters PDAC progression through metabolic imbalances and a hyperglycemic environment.^367^

Inflammation is likewise involved in the process of CFRD. Elevated IL-1β was found in CFRD and non-CF diabetic patients compared to non-CF patients and may cause β cell apoptosis.^368^ Hart et al. found an increase in the inflammatory factors IL-6, IL-1β, CXCL10, TNF-α, and IFN-γ in human CF islets, and these inflammatory factors may cause damage to pancreatic islets in CF patients.^369^ Sun et al. found that CFTR affects β-cell function through paracrine secretion, a mechanism that involves paracrine types of proinflammatory factors.^370^ Whether these inflammatory factors target β cells or are simply part of systemic inflammation remains unknown, but insulin resistance can be induced through acute inflammation.^371^

Inflammation plays a key role in both endocrine and exocrine diseases of the pancreas. Inflammatory factors such as TNF-α and IL-1β not only play a role in the occurrence and progression of pancreatitis,^372^ but also contribute to the development of diabetes by inducing apoptosis and dysfunction of islet cells.^357^ For example, IL-1β increases the risk of diabetes by interacting with insulin signaling pathways, resulting in insulin resistance.^373^ In addition, the persistent inflammatory environment caused by chronic pancreatitis can activate oncogene and tumor suppressor gene mutations, thereby promoting the development of PDAC.^374^

#### Metabolic disturbance

Amino acid dysregulation may be one of the mechanisms contributing to the pathogenesis of CP-related DM. Alterations at the amino acid level are present in CP patients. On the one hand, alterations in the concentration of amino acids may lead to disorders of gluconeogenesis and glycogen synthesis. On the other hand, amino acids are an important regulator of the liver-α cell axis, and hyperaminoacidemia can induce glucagon secretion.^375^ Meanwhile, islet culture studies support a role in selectively stimulating α cell proliferation by amino acids.^376^ The presence of the liver-α cell axis, which allows hepatic glucagon resistance to form hyperglucagonemia, promotes the production of certain amino acids, and the produced amino acids, in turn, promote α cells to secrete glucagon.^377,378^ However, significantly lower levels of citrulline, GABA, taurine, and aspartate were observed in CP patients, but no significantly elevated amino acids were found.^379^ The relationship between disturbances in amino acid metabolism and liver-α cell axis and α cell dysfunction in CP patients needs further investigation.

Hormonal abnormalities also contribute to the metabolism disturbance. Pancreatic polypeptide (PP) deficiency in the postprandial state in patients with CP has been confirmed by a number of studies. There is no significant gap in fasting serum PP levels between CP patients and controls.^380^ PP regulates hepatic insulin receptor expression and utilization and can reverse the decrease in insulin receptor utilization in CP,^381^ leading to improved glucose tolerance. PP deficiency can cause abnormal glucose metabolism, which may be associated with hepatic insulin resistance.^382^ The phenomenon can be reversed by intravenous PP injection,^383^ suggesting a possible role for PP in the development of CP-related DM. Glucose stimulates PP secretion within the islets of mice, and PP has the effect of inhibiting glucagon release,^259^ thus PP deficiency may also be responsible for glucagon elevation in CP patients. The function of PP in glucose metabolism and its deficiency in CP patients highlights a potential area of study. Investigating the mechanisms through which PP regulates hepatic insulin receptor expression and its interaction with glucagon could lead to novel approaches to managing CP-DM.

#### CFTR

The effect of CFTR on the secretory role of β cells has always been controversial. The likely explanation is that CFTR expression varies across species, and its secretory functions differ accordingly. Edlund et al. found that CFTR is involved in insulin secretion from human and mouse pancreatic β cells.^384^ Guo et al. found that CFTR is a regulator of insulin secretion from β cells and that β cells with the F508del mutation (the most common mutation in CF) can be rescued with the mutation correcting agent VX- 809 rescue, increasing insulin secretion from mutant β cells.^385^ However, the use of the corrector and activator Lumacaftor/Ivacaftor in patients with the F508del mutation didn’t improve their glucose tolerance and insulin secretion.^386^ Boom et al. found that CFTR is expressed predominantly in α cells in rats.^387^ Whereas it was found that the F508del elevation of glucagon in mice.^388^ White et al. found that CFTR is expressed in less than 1% of β cells in human pancreatic islets.^389^ However, studies found that mutations in CFTR of CFRD patients do not lead to β cell dysfunction, but are associated with islet loss and inflammatory infiltration.^369,370^ However, the precise molecular pathways through which CFTR mutations lead to β-cell dysfunction in CFRD are not fully understood. Investigating the influences of CFTR on β-cell function and the role of proinflammatory factors in this process could provide insights into new treatment strategies.

#### Paraneoplastic phenomenon

Alongside the various causes of β cell impairment and decreased numbers mentioned above, epidemiological, and clinical studies suggest that PDAC-DM is possibly a paraneoplastic phenomenon mediated by DM-causing substances.^301^ Insulin resistance has been implicated in the pathogenesis of PDAC-DM, which is present in patients with PDAC (normal fasting glucose levels) and disappears after surgical resection.^301^ Islet amyloid polypeptide (IAPP) is believed to be involved in this process. IAPP is secreted by islet cells and is capable of causing insulin resistance in skeletal muscle cells.^390^ IAPP levels were significantly elevated in PDAC patients compared to those with other cancers, diabetes, and healthy individuals.^391^ PDAC cells specifically promote islet cell secretion of IAPP.^392,393^ However, IAPP is not an ideal biomarker for the diagnosis and identification of PDAC.^394^ Its molecular mechanism in the development of PDAC-DM remains to be explored. PDAC-derived S-100A8 induces insulin resistance in vitro experiments.^395,396^ This suggests its potential as a biomarker for the early detection of PDAC. And identifying other biomarkers for early detection of PDAC-DM remains a critical area of research.

### Mechanisms of endocrine diseases causing PDAC

#### Islet inflammation

Long-term T2D is often accompanied by chronic inflammation. Hotamisiligil et al. found that TNF-α plays an important role in diabetes and obesity-related insulin resistance, and blocking TNF-α can improve insulin resistance.^397^ Inflammatory markers CRP, IL-6, and leptin levels in diabetic patients were significantly higher than in non-diabetic patients, while adiponectin was lower than in non-diabetic patients.^398^ Increasing evidence suggests that chronic inflammatory responses play a role in the pathogenesis of insulin resistance, with adipose tissue being an important source of pro-inflammatory cytokines.^399^ In pancreatic tissue, this is often manifested as cytokines, apoptotic cells, immune cell infiltration, chronic inflammatory infiltration, and amyloid protein deposition, ultimately leading to fibrotic chronic inflammation. IL-1β plays a crucial role in this process.^400^ The chronic inflammatory microenvironment in the pancreas may be one of the reasons contributing to the occurrence and development of PDAC. However, the involvement of inflammatory mediators like IL-1β, TNF-α, and IFN-γ in pancreatic diseases and their effect on β-cell dysfunction needs more in-depth study. Specifically, how these mediators interact with pancreatic cells and contribute to the progression of diseases such as PDAC and pancreatitis warrants further research.

#### Obesity

Obesity is closely related to diabetes.^401^ Overweight or obesity is a crucial predictor of diabetes. In one cohort study, the risk of diabetes increased by 20.1 times when the Body Mass Index (BMI) of women was in the range of 30.0–34.9 kg/m^2^, and when BMI was ≥35 kg/m^2^, the risk increased by 38 times.^402^ For men aged 25–49 years old with a BMI between 30.0 and 34.9 kg/m^2^, the risk of diabetes was 10.1 times higher than that of normal-weight men.^403^ However, more and more research suggests that an increase in visceral fat measured by CT and MRI at any BMI level will lead to an increased risk of diabetes.^404^ At the same time, overweight and obesity are found to be closely related to an elevated risk of PDAC and other cancers.^405^ Compared to lean individuals, obese individuals have a 47% higher incidence of PDAC.^406^ In a normal pancreas, obesity can induce inflammation and fibrosis. In mice, adipocytes are able to secrete IL-1β, recruit tumor-associated neutrophils, and induce the activation of PSC, thereby promoting the proliferation of connective tissue, accelerating tumor growth, and weakening the therapeutic effect of chemotherapy drugs.^407^

As an endocrine tissue, adipose tissue can secrete adipokines, many of which can promote inflammation.^408^ While some adipokines are produced due to immune cell infiltration in adipose tissue, leptin, and adiponectin are adipose tissue-specific adipokines, and they may be both involved in the pathophysiological mechanisms of PDAC.^409^ Leptin is a product of the obese gene expression and has been proven to be related to various inflammatory and immune responses.^410^ Leptin participates in the pathophysiological mechanisms of obesity-related diseases such as T2D and cancer by activating the PI3K/mTOR pathway.^411^ Meanwhile, leptin may promote glucose metabolism and cell proliferation in PDAC cells by activating the AKT pathway.^412^ Fan et al. found that leptin triggered the migration and invasion of PDAC by upregulating matrix metalloproteinase-13 (MMP-13).^413^ Adiponectin is an adipokine that acts on various tissues and participates in homeostatic regulation.^414,415^ Adiponectin also inhibits PDAC cell apoptosis, increases PDAC cell proliferation, and promotes migration through the activation of the AMPK/Sirt1/PGC1-α signaling pathway.^416^

#### Antidiabetic drugs and PDAC

Metformin (which lowers glucose and insulin levels), sulfonylureas (which promotes insulin secretion from the pancreas), and insulin analogs (such as glargine insulin) are therapeutic options for diabetes. A case-control study revealed that metformin significantly lowered the risk of PDAC in diabetic patients, whereas the use of insulin or insulin secretagogues was linked to a higher PDAC risk in individuals with diabetes.^417^

A retrospective study found that metformin significantly improves the two-year survival rate of PDAC patients with diabetes.^418^ KisfalviK et al. discovered that in a mouse xenograft model of PDAC, metformin significantly inhibits tumor growth. This inhibition may be due to metformin disrupting the signaling crosstalk between IR and GPCRs.^419^ Subsequently, they found that this effect is dose-dependent, and oral administration of metformin before and after tumor implantation significantly inhibits the growth of PDAC xenografts.^420^ In genetically engineered mice, intraperitoneal injection of metformin inhibits the occurrence of PDAC by suppressing the NFκB/STAT3 signaling pathway.^421^ In mice, metformin inhibits ADM and mouse pancreatic intraepithelial neoplasia (mPanIN), thereby suppressing pancreatic carcinogenesis.^422^ Meanwhile, Chang et al. found that metformin inhibits KrasG12D-induced ADM, PanIN, hyperinsulinemia, and hyperleptinemia induced by a high-fat, high-calorie diet, thereby inhibiting the occurrence of PDAC.^423^

Mechanistically, metformin’s anticancer effects may be both direct, targeting PDAC cells, and indirect, via systemic impacts on the pancreas.^424^ At the cellular level, activation of AMPK inhibits mTOR, ERK activation, and DNA synthesis, and concurrently inhibits YAP /TAZ to suppress PDAC development.^425^ Wang et al. found that metformin inhibits PDAC metastasis by inhibiting the SMAD4-HNF4G pathway.^426^ In addition, it exerts anti-tumor effects by lowering glucose, insulin, and IGF levels and normalizing the gut microbiota in diabetic/obese PDAC patients.

Chang et al. found that the use of glargine insulin increases the risk of PDAC in male diabetic patients.^427^ A meta-analysis identified a positive association between the use of insulin and insulin analogs and the incidence of PDAC.^428^ Insulin acts as a growth-promoting factor with a mitogenic effect on cells, promoting cell proliferation in both normal and cancer cells.^429,430^ Possible reasons include the activation of IGF-1R and MAPK pathways by insulin and insulin analogs.^431^ Wu et al. found that insulin promotes PDAC cell proliferation and migration by activating the PI3K/Akt pathway.^136^ In addition, insulin enhances PDAC cell lines’ PANC-1 proliferation and invasion by stimulating the HIF-1α pathway.^432^

More basic and epidemiological research is needed to understand the mechanisms between anti-diabetic drugs and PDAC. This understanding can guide diabetic patients in rational drug use, reducing the potential cancer risks associated with diabetes treatment. Additionally, it contributes to the discovery of molecular therapeutic targets for PDAC in diabetic patients.

#### Hyperglycaemia

A study indicated an increased risk of PDAC associated with elevated fasting blood glucose levels.^433^ High blood glucose induces the activation of p38 MAPK, mediating the paracrine secretion of IL-6 and VEGF, thereby promoting the proliferation and migration of PDAC cells.^433^ Glucose dosage dependently increases the expression of glial cell line-derived neurotrophic factor (GDNF) and its tyrosine kinase receptor (RET), promoting the growth of PDAC cells.^434^ Prolonged hyperglycemia leads to the accumulation of advanced glycation end products (AGE). The binding of AGE to its receptor (RAGE) can stimulate the secretion of pro-inflammatory factors, and generate oxidative stress and reactive oxygen species, ultimately leading to the activation of NF-κB and target genes, contributing to carcinogenesis.^435^ In KC mouse, the deletion of the RAGE inhibits the occurrence and progression of PDAC and extends survival,^436^ suggesting that AGE and RAGE have the potential to serve as therapeutic targets for PDAC. Further research is needed to understand their precise roles and mechanisms in PDAC.

#### Hyperinsulinemia

Obesity and diabetes are often accompanied by insulin resistance, manifested as increased levels of serum insulin and decreased sensitivity of tissues to insulin. In this condition, the liver’s uptake of glucose is reduced, and peripheral tissues also exhibit decreased glucose uptake, resulting in increased circulating glucose levels. This stimulates β cells to secrete insulin, leading to compensatory hyperinsulinemia. Both hyperinsulinemia and hyperglycemia are considered risk factors for increased incidence and mortality of PDAC.^437^ Bao et al. found that dietary insulin load was not correlated with PDAC occurrence in healthy individuals, but a high insulin-load diet might increase the cancer risk in individuals with insulin resistance.^438^ Elevated insulin levels may increase the replication rate of PDAC cells in the early stages.^439^ Conversely, a diet with a low insulin load can reduce the incidence of PDAC.^440^ In obese mice induced by a high-fat diet, there is a significant increase in serum insulin secretion and a marked increase in the proliferation of PDAC cells.^441^ Zhang et al. suggest that endogenous insulin, independently of high blood glucose, facilitates the progression of PanIN in a mouse model of a high-fat diet.^442^

Insulin acts on the IR, and upon binding, it can activate downstream MAPK and PI3K pathways, thereby promoting cell proliferation.^443^ High concentrations of insulin can dose-dependently stimulate the proliferation of PDAC cell lines.^444^ Insulin plays a potential role in promoting the proliferation and survival of PDAC cell lines, more dependent on the RAF/Erk signaling pathway.^445^ Recent studies indicate that insulin, in a dose-dependent manner through the insulin receptor, increases the secretion of pancreatic enzymes and the formation of ADM, thereby promoting the development of PDAC.^294^ On the other hand, the formation of PDAC is accompanied by fibrosis in pancreatic stromal tissue. Insulin can promote the growth of PSC and the occurrence of stromal fibrosis.^446^ Overexpression of docking peptides observed in human PDAC tissues and cell lines results in the activation of intracellular IR, IRS1, and IRS2, leading to excessive activation of the PI3K signaling cascade.^447–449^ In PDAC, IR has been found to undergo G protein-coupled receptor cross-talk, activating mTOR, thereby stimulating cancer cell DNA synthesis and proliferation.^450^ Additionally, it can stimulate YAP localization through PI3K and PKD pathways, promoting the growth of PDAC cells.^451^

#### IGF system

In T2D, increased insulin secretion leads to enhanced bioavailability of IGF.^452^ Under physiological conditions, IGF is secreted by the liver and acts on IGF-1R, playing a role in inhibiting apoptosis and promoting proliferation.^453,454^ In patients with PDAC, the expression of IGF-1R is upregulated and correlates with higher tumor grades and shorter overall survival.^455,456^ Mutated Kras and downstream MAPK pathways, combined with autocrine activation of IGF-1R by IGF-2, play a critical role in triggering PI3K signaling and driving the proliferation of PDAC cells.^457^ IGF-1 can also stimulate the proliferation of PDAC cells, and this effect can be significantly suppressed by antibodies against IGF-1R.^458^ However, clinical trial results with IGF-1R antibodies have been less satisfactory.^459^ One possible reason is the presence of IR in PDAC with a similar structure and function to IGF-1R. They are both transmembrane receptor tyrosine kinases from the same family. In normal tissues, IR is more involved in glucose metabolism, while IGF-1R is more responsible for cell proliferation and anti-apoptosis.^120^ IR also has two isoforms: IR-A, mainly expressed in embryonic and adult brain tissues, and IR-B, mainly expressed in well-differentiated adult tissues, enhancing the effects of insulin. IR-A is overexpressed in tumor cells, heightening their sensitivity to IGF-2 and insulin, and contributing to tumorigenesis.^120,460^ In cancer tissues, hybrid receptors IR/IGF-1R exist, especially IR-A/IGF-1R, which has a high affinity for IGF2, enhancing cellular responses to mitogenic signals. All these findings indicate that IR/IGFR could be a potential therapeutic target in PDAC. Further research on the exact functions of IGF1R and IR in PDAC, including their specific roles in cancer cell growth, differentiation, and migration, will contribute to a more comprehensive understanding of their roles in disease development and the development of related drugs.

**Fig. 6:**
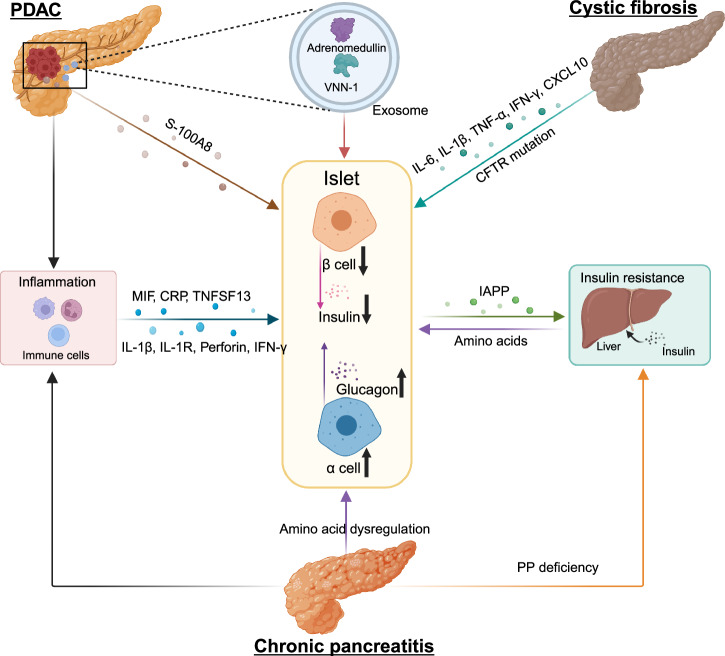
Mechanisms of PDAC-DM, CP-related DM, and CFRD. Inflammatory microenvironment is present in PDAC, CP, and CF. These diseases stimulate immune cells in the microenvironment to secrete various cytokines that act on β cells, causing β cell death or dysfunction. PDAC cells act on β cells through exosomes or their own secretions, causing them to be dysfunctional. β cells can secrete IAPP, mediating insulin resistance. CP can lead to amino acid disorders, resulting in abnormal numbers and dysfunction of β and α cells. CP can also mediate insulin resistance by reducing PP production. Some amino acids secreted by the liver act on α cells to secrete glucagon. The abnormal number and dysfunction of α and β cells is an important mechanism for the formation of diabetes mellitus. Created in BioRender.com

Here we briefly describe the interaction between the endocrine and exocrine pancreas from the perspective of disease development. (Fig. 6) Mechanistically, pancreatic exocrine diseases can impact islet cells through various pathways, contributing to the dysfunction and reduction in the number of β cells, ultimately leading to the development of diabetes. Conversely, diabetes can influence the development of PDAC through factors such as islet inflammation, obesity, hyperinsulinemia, and hyperglycemia. The interaction between the endocrine and exocrine systems is particularly important in pancreatic diseases. In the occurrence and development of diseases such as pancreatitis and diabetes, the interaction of endocrine and exocrine functions often suggests the potential interaction between the endocrine and exocrine pancreas. Such interactions not only reveal the complex regulatory network of endocrine and exocrine but also hint at the potential mechanisms by which these systems interact in disease states. This crosstalk between pancreatic endocrine and exocrine diseases provides a new perspective for us to understand the full picture of pancreatic disease and may provide new targets for clinical treatment. However, more studies are needed about the inter- and intracellular crosstalk between the endocrine and exocrine pancreas.

## Clinical implication and therapeutic targets

In recent years, advances in medical research have highlighted the critical role of signaling pathways in health and diseases. Among these pathways, the importance of pancreatic endocrine and exocrine signaling pathways in a variety of diseases has become increasingly prominent, providing new hope and direction for clinical treatment. An in-depth understanding of its complex regulatory mechanisms and potential therapeutic targets is of great significance for clinical practice.

### Diabetes

The insulin signaling pathway plays an important role in a variety of metabolic and endocrine diseases. The abnormal function of insulin receptors and their subsequent signaling molecules is a key pathological feature of T2D.^461^ Increasing evidence indicates that mutations and dysregulation in key genes within the insulin signaling pathway contribute to the development of diabetes. For instance, mutations in the IR gene have been linked to various severe insulin resistance conditions, such as Leprechaunism, Rabson–Mendenhall syndrome, and Type A insulin resistance syndrome.^462^ In cases of insulin resistance, the efficiency of insulin signaling is reduced, resulting in decreased glucose uptake, diminished glycogen synthesis in the liver, and increased lipolysis in adipocytes, which subsequently triggers hyperglycemia and other metabolic disorders. The required insulin levels in these patients are a hundred times higher than those in typical diabetes patients, and Nonsense or missense mutations have been detected in either the extracellular insulin-binding domain or the intracellular tyrosine kinase domain of their receptors.^463^

Mutations in the IRS1 gene are closely linked to insulin resistance.^464^ In patients with T2D, the G972R polymorphism of IRS-1 is observed with higher frequency, leading to reduced insulin signaling, primarily due to decreased PI3K activity.^465^ Further studies have shown an association between IRS-1 mutations and SNPs in T2D.^466^ Mice with an Irs-1 gene knockout exhibit growth retardation and insulin signaling defects.^467^ Phosphorylation site mutations in IRS1 can disrupt the interaction with IR, thereby weakening PI3K activation, resulting in impaired AKT signaling. This signaling impairment prevents insulin from effectively promoting glucose uptake, leading to elevated blood glucose levels, a condition particularly common in patients with T2D.^468^

In addition to IRS1, mutations in the AKT gene are another significant cause of insulin signaling pathway dysregulation. A rare missense mutation (R274H) in AKT2 found in diabetes patients results in the loss of kinase activity.^469^ Although other missense mutations (such as R208K and R467W) do not cause a loss of kinase activity in vitro,^470^ functional loss mutations in AKT can impede the normal activation of its downstream targets, mTORC1 and GSK-3β, leading to reduced glycogen synthesis and impaired glucose uptake.^471^ Furthermore, dysregulation of AKT signaling is associated with increased lipolysis in adipocytes, which exacerbates insulin resistance and hyperglycemia.^472^

PI3K mutations are also key factors leading to insulin signaling dysregulation. While activating mutations in the PI3K gene are common in various metabolic diseases and cancers, which may result in metabolic disorders, in the context of diabetes, its dysfunction is often characterized by abnormally weakened downstream AKT signaling. The M326I polymorphism of the PI3K p85α regulatory subunit, identified in Pima Indian women, is associated with a reduced incidence of T2D.^473^ However, this mutation adversely affects insulin signaling by reducing the binding of p85α to IRS-1 and increasing p85α degradation.^474^

Drugs such as insulin and its analogs that target insulin receptors and their subsequent signaling molecules for the treatment of diabetes have shown good anti-diabetic effects in practical clinical applications.^475^ Although the mechanisms of insulin signaling pathway dysregulation in diabetes have been extensively studied, many details remain to be explored. In particular, how to effectively restore key nodes in the insulin signaling pathway to prevent or reverse the progression of diabetes continues to be a significant research focus. Targeting key molecules within the insulin signaling pathway may offer new opportunities for diabetes treatment. These therapeutic opportunities depend on individualized genetic mutations, suggesting that precision medicine strategies based on individual genetic mutations could be an important direction for future diabetes treatment.

The role of glucagon is more pronounced due to insufficient insulin production. Glucagon activates a signaling pathway through its receptors (expressed primarily in the liver) that promotes glycogenolysis and gluconeogenesis, resulting in elevated blood glucose levels.^476^ This is one of the important mechanisms of hyperglycemia in diabetic patients. In T2D, although insulin resistance is the main cause, glucagon secretion is often abnormally increased, exacerbating the difficulty of blood glucose control.^477^ Increasing evidence demonstrates that inhibiting glucagon and its receptor can reduce hyperglycemia in both animal models and humans, underscoring the pivotal role of glucagon and GCGR in diabetes development.^182,478^ In GCGR knockout (Gcgr−/−) mice, during complete insulin deficiency, glucagon-inhibitory factors like somatostatin can suppress all metabolic manifestations of diabetes, indicating that β-cell destruction does not lead to diabetes.^479^ When streptozotocin was used to destroy β-cells in Gcgr−/− mice and inhibit insulin secretion, the animals did not develop hyperglycemia, indicating that Gcgr−/− mice do not develop T1D, even without insulin.^480^ Temporary restoration of defective GCGR using an adenoviral vector led to elevated blood glucose levels after β-cell destruction.^481^ In insulin tolerance tests, these knockout mice exhibited enhanced glucose tolerance and insulin sensitivity.^482^ The elevated blood glucose levels resulting from insulin deficiency were normalized when glucagon was eliminated. This is an unexpected result as it underscores the significant potential of GCGR in diabetes treatment. Mechanistically, glucagon’s regulation of blood glucose depends on β cells, as glucagon acts on β cells to activate downstream molecules, leading to increased cAMP production, which is closely related to insulin release by β cells.^483^ In fact, mice lacking GCGR develop hyperinsulinemia and compensatory hyperplasia of α-cells. This limits the role of GCGR gene knockout in the treatment of diabetes in rodents, as it triggers a range of other metabolic issues. GCGR is considered a candidate gene in the pathogenesis of T2D. In a study of the French diabetic population, the frequency of the Gly40Ser mutation in the GCGR gene was found to be as high as 5%, far exceeding that of other genes.^484^ An analysis of 64 diabetic children in China suggested that the Gly40Ser mutation in the GCGR gene may be associated with genetic susceptibility to T2D,^485^ as it disrupts the binding of GCGR to glucagon.^486^ It is hypothesized that the Gly40Ser mutation in GCGR may contribute to β cell dysfunction and increase the risk of diabetes by disrupting glucagon signaling and decreasing the sensitivity of target tissues to glucagon.

Targeting GCGR with antagonists, including small molecule inhibitors and monoclonal antibodies, has been suggested as a potential therapeutic strategy for managing both T1D and T2D.^487–489^ Several GCGR antagonists, such as MK-0893, MK-3577, LY2409021, and LGD-697, have been developed to enhance glucose tolerance, boost insulin secretion, and regulate blood glucose levels in animal models, demonstrating considerable efficacy in patients with T2D.^490–493^ GCGR monoclonal antibodies (mAbs) exhibit high specificity and strong targeting capabilities, making them relatively accessible. They not only normalize blood glucose levels in insulin-naive T1D mice and patients but also show potent hypoglycemic effects in T2D mice and monkeys.^494–496^ GCGRmAbs were also able to induce δ-cell proliferation and transdifferentiation into β cells along with ductal cells.^497^ Also, anti-GCGR antibodies were shown to promote β cell proliferation and α cell transdifferentiation into β cells.^498^ The underlying molecular mechanisms remain to be explored. Pathologically, glucagon can induce β cell dedifferentiation, leading to loss of function, while GCGR mAbs can reverse this process by reducing FoxO1 expression.^499^

### Non-alcoholic fatty liver disease

In non-alcoholic fatty liver disease (NAFLD), insulin resistance leads to abnormal accumulation of fat in the liver, which further triggers inflammation and liver cell damage, eventually leading to cirrhosis and liver failure.^500^ The IR/PI3K/Akt signaling pathway is crucial for the development of NAFLD. A genome-wide association meta-analysis identified the INSR gene as being associated with NAFLD.^501^ Polymorphisms in the INSR gene are significantly correlated with the occurrence of NAFLD, with some INSR gene polymorphisms exerting a protective effect against NAFLD. Mice with liver-specific insulin receptor knockout exhibit marked insulin resistance.^502^ Liver-IRKO mice display reduced levels of plasma IGF-1, delayed early growth, and disruptions in fatty acid metabolism, including lower expression of lipogenic enzymes and plasma triglycerides.^503^ Mice with liver-specific Irs1/Irs2 double knockout (LIrs1/2DKO) show glucose intolerance, hyperinsulinemia, and impaired regulation of hepatic glucose production, while Irs2KO mice develop liver steatosis.^504^ While Akt1 is vital for cell survival and growth, Akt2 plays a more crucial role in the liver.^113^ Mice deficient in hepatic Akt1 and Akt2 display abnormal glucose tolerance, insulin resistance, and impaired transcriptional responses to feeding, including reduced expression of lipogenic genes,^505^ which may trigger increased liver fat production.

Liver-specific PTEN-knockout (LPTENKO) mice develop severe hepatomegaly and steatohepatitis with triglyceride accumulation, alongside elevated expression of lipogenic genes and increased β-oxidation.^506–508^ Although an increased incidence of hepatocellular carcinoma was also observed, LPTENKO mice not only showed improved glucose tolerance but also enhanced systemic insulin sensitivity due to the redistribution of fat from adipose tissue to the liver and/or increased hepatic FGF21 production. Profound alterations in hepatic lipid metabolism occur in the context of insulin resistance.

In NAFLD, dysregulation of the glucagon signaling pathway is a key pathological factor, especially in the context of insulin resistance. This dysregulation is primarily manifested as hepatic glucagon resistance. In NAFLD, despite elevated levels of glucagon in the blood, the liver’s response to glucagon is diminished. This glucagon resistance leads to disruptions in lipid and amino acid metabolism, resulting in excessive fat accumulation in the liver, increased oxidative stress in hepatocytes, and ultimately, the progression of NAFLD.^509^ Therapeutic strategies targeting this signaling pathway, such as the use of glucagon receptor antagonists, may offer new treatment avenues for the management of NAFLD. However, the specific mechanisms of glucagon signaling dysregulation in NAFLD require further investigation, particularly its precise role in hepatic lipid metabolism and inflammatory responses. Understanding these mechanisms will aid in the development of more effective therapeutic strategies that target various aspects of the glucagon signaling pathway, alleviating the hepatic burden in NAFLD patients and improving disease outcomes.

### Cancer

The role of insulin signaling in cancer has also received much attention. High insulin levels and over-activation of IGF are associated with the development and progression of certain cancers, such as breast, colon, and prostate cancers, and may promote tumor growth by promoting cell proliferation and inhibiting apoptosis. Research indicates that hyperinsulinemia is associated with an increased risk of certain types of cancer.^510^ Studies have demonstrated that overexpression of insulin resistance in certain tumor cell lines boosts their proliferative response to insulin.^511^ Furthermore, insulin has the ability to stimulate the elevated levels of many proliferation and differentiation regulators, including IGF-1, cytokines, and growth factors such as leptin, VEGF, and IL-6.^512–514^ These effects may drive tumor progression, angiogenesis, and metastasis, explaining how hyperinsulinemia and insulin resistance raise the risk of developing cancers like breast, colon, liver, pancreatic, and endometrial cancers.^515–517^ Given these mechanisms, inhibitors targeting the insulin receptor have been developed for the treatment of various cancer.^518^ Several drugs targeting IR/IGF-1R signaling have shown anti-tumor growth in tumor xenotransplantation models, but their effectiveness in prostate cancer patients needs to be further validated in clinical trials.^519^ Gene mutations in some key molecules of the insulin receptor signaling pathway, such as PI3K and PTEN, have been found to be closely related to cancer development, as has been well-reviewed by several scientists.^520–522^ Targeted drugs against these signaling molecules, such as PI3K inhibitors, have shown some promising results in clinical trials.^520^ However, due to the significant side effects and poor solubility and permeability of these drugs, obtaining FDA approval has been challenging. When developing new drugs, designing highly specific drugs with fewer side effects based on mutation sites is a major hurdle that needs to be addressed in the current development of this class of therapeutics.

In many cancers, particularly neuroendocrine tumors, the expression of SSTRs is significantly reduced.^523^ SSTR2 is the most studied receptor, primarily inhibiting tumor growth by suppressing cell proliferation signaling pathways (such as PI3K/AKT and MAPK) and activating apoptotic pathways (such as p53). However, in many cancers, the expression of SSTR2 is reduced or lost, diminishing the antitumor effects of somatostatin.^524,525^ This reduction leads to decreased efficacy of somatostatin and its analogs in treatment. Even when SSTRs are expressed in cancer cells, tumor cells may evade inhibition by altering downstream signaling pathways.^239^ For instance, in some tumors, SSTR activation may fail to effectively initiate inhibitory signals, or downstream pathways such as PTP, PI3K/AKT, and MAPK may be altered by other mutations or regulatory mechanisms, significantly reducing the antiproliferative effects of SSTRs.^243,526^ Due to the dysregulation of SSTR expression and function, therapeutic strategies targeting these receptors (such as using octreotide, lanreotide, and other SSTR analogs) are limited in effectiveness for certain cancers. This dysregulation suggests the need for new strategies, which may include combination therapies or the development of drugs targeting other somatostatin receptor subtypes.

### Neurological disorders

Research shows that brain insulin resistance, or the disruption of insulin signaling in the brain, is linked to cognitive decline and Alzheimer’s disease (AD), making it a risk factor for sporadic AD development. The hallmark AD pathologies, such as neurofibrillary tangles (NFTs) and amyloid-beta plaques, may be connected to insulin resistance.^527^ Insulin, along with IGF-1 and IGF-2, can prevent neuronal apoptosis through the IR and regulate neurobiological processes, including the degradation of amyloid-beta.^528,529^ However, amyloid-beta can compete with insulin for IR binding, reducing insulin’s affinity for IR and leading to insulin resistance, which in turn worsens AD pathology.^530^ Increased serine phosphorylation of IRS-1, dysfunction in the PI3K pathway, and AKT inhibition activate GSK-3β and mTOR phosphorylation, contributing to excessive tau protein phosphorylation. Additionally, GSK-3β activation promotes amyloid-beta plaque buildup, potentially triggering microglia-mediated neuroinflammation that drives AD progression.^531–533^ Activation of the Ras-MAPK pathway further enhances gene transcription related to neuronal survival, synaptic plasticity, and tau phosphorylation, which is also linked to amyloid-beta accumulation and NFT formation.^534^ The mechanisms outlined above provide potential therapeutic strategies for targeting insulin signaling pathways in AD treatment. Future research should continue to explore the specific roles of these signaling pathways in AD and develop effective therapies targeting these pathways.

Somatostatin also plays an important role in the nervous system by regulating the release of neurotransmitters and the excitability of nerve cells and is involved in the regulation of various neural functions.^535^ In certain neurological disorders such as Alzheimer’s disease abnormalities in somatostatin signaling pathways may affect the progression of the disease and the manifestation of symptoms.^536,537^ In AD, the reduced expression of SSTR is associated with increased neuronal apoptosis and cognitive decline. The loss of SST and SST-expressing neurons in AD is a well-established event.^538^ The reduction in somatostatin signaling may lead to decreased neuronal resistance to excitotoxicity and oxidative stress, thereby accelerating the progression of AD pathology. A unique feature of SST is its ability to enhance the activity of Aβ-degrading enzymes in the brain through receptor-mediated actions.^539^ Studies have shown that activation of SSTR2 can inhibit the production and deposition of amyloid-beta, suggesting that restoring SSTR2 function may have protective effects against AD.^536,540^ Somatostatin-based therapeutic strategies could offer new approaches to slowing the progression of AD. However, due to the complex pathology of AD, treatments targeting a single pathway may have limited efficacy. Future research could explore combining SSTR agonists with other neuroprotective agents to achieve multifaceted protective effects.

### Digestive diseases

Somatostatin signaling plays an important role in digestive diseases. Somatostatin regulates the digestive process by inhibiting gastric acid secretion, pancreatic enzyme secretion, and intestinal peristalsis. In acute pancreatitis, somatostatin helps reduce inflammation and damage to the pancreas by reducing pancreatic enzyme secretion and pancreatic load.^541^ Somatostatin, as a pancreatic enzyme inhibitor, has been widely used in patients with acute pancreatitis.^541^

The cholecystokinin (CCK) signaling pathway plays an important role in a variety of digestive diseases. For example, abnormal activation of calcium signaling pathways in acute pancreatitis leads to premature activation of pancreatic enzymes and digestion by the pancreas itself.^542^

In chronic pancreatitis, persistent activation of CCK signaling is associated with pancreatic fibrosis and loss of acinar cells, ultimately leading to reduced digestive enzymes and impaired nutrient absorption.^543^

An antagonist targeting the CCK receptor has shown beneficial effects in a Phase I clinical trial of pain management in patients with chronic pancreatitis.^544^

CCK also plays a key role in cholelithiasis and gallbladder diseases, affecting the emptying of the gallbladder and the function of the biliary system through its effect on the smooth muscle of the gallbladder.^545^ Thus, CCK receptor (primarily CCK1 receptor) antagonists are being investigated as potential agents for the treatment of cholelithiasis, gallbladder disease, and certain gastrointestinal disorders. These drugs can block the binding of CCK to its receptors, thereby regulating the emptying of the gallbladder and the function of the biliary system, and may have certain effects on the treatment of the disease.

In functional gastrointestinal disorders such as indigestion and irritable bowel syndrome,^546^ CCK signaling regulates the movement and secretion of the gastrointestinal tract,^547^ and abnormal CCK signaling may contribute to the symptoms of these disorders. Thus, the CCK receptor may act as a potential therapeutic target of these disorders.

### Cardiovascular disease

Insulin resistance is closely associated with atherosclerosis and hypertension, accelerating arteriosclerosis and cardiovascular events by triggering endothelial cell dysfunction and promoting inflammatory responses.^548^ And the role of glucagon cannot be ignored in cardiovascular disease. Through its receptors in the heart and blood vessels, glucagon regulates myocardial contractility and vascular tone, affecting blood pressure and heart function.^549^ Abnormal glucagon signaling may increase the risk of atherosclerosis. However, endocrine signaling has not been used as a therapeutic target for the treatment of cardiovascular disease.

In conclusion, the exploration of pancreatic endocrine and exocrine signaling pathways in the pancreas opens up new ways to treat a variety of diseases. From metabolic diseases to tumors, targeting pancreatic endocrine and exocrine signals offers promising strategies for alleviating symptoms and improving prognosis. Potential therapeutic targets for these diseases and drugs in clinical trials are listed in table 1. As research further reveals its complexity, the prospect of harnessing the pancreatic endocrine and exocrine signaling pathways for therapy remains an exciting new area in medical science.

**Table 1:** Clinical trials targeting endocrine and exocrine signaling across various disorders

Compound	Therapeutic targets	NCT number	Phase	Enrolled diseases	Status
64Cu-DOTATATE	SSTR	NCT03673943	3	Neuroendocrine Tumors	Completed
Pasireotide	SSTR	NCT01620138	2/3	Non-functioning Pituitary Adenomas, Prolactinomas	Completed
Octreotide	SSTR	NCT00595140	4	Acromegaly	Completed
Indium-111 pentetreotide	SSTR	NCT00442533	2/3	Neuroendocrine Tumors	Completed
177Lu-edotreotide	SSTR	NCT05918302	3	Neuroendocrine Tumors, Lung Neuroendocrine Neoplasm, Thymus Neoplasms	Recruiting
PP	PP receptor	NCT03854708	4	Hunger	Completed
PP	PP receptor	NCT00791076	2	T1D	Terminated
PP1420	PP receptor	NCT02221765	2	Obesity	Terminated
PP1420	PP receptor	NCT02221765	1	Obesity	Completed
RG1068 (Synthetic Human Secretin)	Secretin receptor	NCT00036231	3	Autism and gastrointestinal disorders	Terminated
ChiRhoStim	Secretin receptor	NCT01087801	3	Pancreatic Disease	Completed
Insulin	IR	NCT03899402	2/3	T1D	Recruiting
Figitumumab	IGF-1R	NCT00977561	2	Small Cell Lung Carcinoma	Terminated
Figitumumab	IGF-1R	NCT00927966	1	Sarcoma Solid Tumor	Completed
Figitumumab	IGF-1R	NCT00147537	1/2	Advanced Lung Cancer	Completed
MK0646	IGF-1R	NCT00610129	2	Neuroendocrine Tumors, Metastatic Neuroendocrine Tumors	Completed
MK0646	IGF-1R	NCT00769483	1/2	Pancreatic Cancer	Completed
MK0646	IGF-1R	NCT00769483	1/2	Lung cancer	Completed
AMG479	IGF-1R	NCT01024387	2	Neuroendocrine Tumor, Carcinoid Tumor, Pancreatic Neuroendocrine Tumor	Completed
IMC-A12	IGF-1R	NCT00785538	1	Advanced Solid Tumors	Completed
BIIB022	IGF-1R	NCT00555724	1	Solid Tumors	Completed
R1507	IGF-1R	NCT00400361	1	Neoplasms	Completed
LY2409021	Glucagon receptor	NCT01606371	1	T2D	Completed
PF-06293620	Glucagon receptor	NCT02211261	1	T2D	Completed
Dasiglucagon	Glucagon receptor	NCT03667053	3	Hypoglycemia	Completed

## Conclusion and perspective

The dual functions of the pancreas encompass both endocrine and exocrine functions, highlighting its critical role in maintaining metabolic and digestive homeostasis. The complex signaling in the endocrine and exocrine systems plays an important role in the occurrence and progression of various diseases. This review summarizes key historical milestones of pancreatic components, embryonic development and phenotypic transformation, and the signaling pathways involved in both health and disease states. By elucidating the signaling and their regulatory of endocrine and exocrine pancreas, we have gained a deeper understanding of the complex regulatory networks within the pancreas. These insights provide new directions for innovative treatments for endocrine and exocrine dysfunction and offer new hope for patients with various diseases.

However, current studies have often focused on one system in isolation, which limits our understanding of how these systems interact in disease contexts. Technical challenges, such as difficulties in tracking real-time cellular interactions and limited tools to study both endocrine and exocrine pathways concurrently, have hindered progress in this area. Another significant challenge lies in the limitations of current research models. While animal models, particularly in mice, have been useful in uncovering the basic mechanisms of pancreatic development and function, translating these findings to humans has proven difficult due to species-specific differences in gene expression, developmental timing, and cellular behavior. Furthermore, disease heterogeneity is another major hurdle. Both PDAC and diabetes exhibit significant variability not only between patients but also within different regions of the same pancreas. This heterogeneity complicates efforts to identify consistent biomarkers or therapeutic targets, as signaling pathways may behave differently depending on the specific pathological context.

In addition, although these structural and functional features suggest complex interactions between endocrine and exocrine pancreas, these interactions have in fact been poorly studied. In this review, the interaction of pancreatic endocrine and exocrine diseases was discussed from the perspective of the pathogenesis and development of pancreatic diseases. Especially in the process of pancreatic diseases, whether the changes of endocrine and exocrine functions will directly or indirectly affect each other is still a field to be further studied. We need to learn more about the interaction between the endocrine and exocrine pancreas, especially the direct cell-cell interaction. And whether this interaction can lead to the occurrence and development of disease, remains to be explored. Regarding the signaling of endocrine and exocrine pancreas, we know that these signals not only regulate the function of the pancreas but also have many other roles. However, whether there is crosstalk between endocrine and exocrine signaling pathways and whether this crosstalk mediates the occurrence of diseases is also an important direction worthy of further exploration.

Emerging technologies such as advanced imaging, single-cell RNA sequencing, and spatial transcriptomics offer promising avenues for overcoming current limitations. These techniques allow for the visualization of cellular interactions and the identification of cellular heterogeneity within the pancreas, potentially revealing novel therapeutic targets. Future studies should continue to explore the molecular basis of both the intra- and intercellular pathways of pancreatic interactions and to explore the dynamic changes in the islets’ microenvironment during PDAC, identify early biomarkers for alterations in hormones of PDAC, and utilizing advanced imaging and single-cell technologies to uncover cellular heterogeneity and plasticity within the pancreas. Understanding how the microenvironment within islets changes during disease progression may provide early biomarkers for detecting pancreatic dysfunction before it becomes symptomatic. Additionally, investigating the systemic effects of pancreatic dysfunction on other organs could lead to a more comprehensive understanding and treatment of pancreatic diseases, broadening therapeutic approaches, and offering holistic treatments for conditions like diabetes and pancreatic cancer. Overall, exploring the crosstalk between endocrine and exocrine of the pancreas and their role in diseases will provide novel insights, help better understand the pathogenesis of various diseases, and provide a theoretical basis for future research and treatment strategies.
